# Evolutionary interactions between haemagglutinin and neuraminidase in avian influenza

**DOI:** 10.1186/1471-2148-13-222

**Published:** 2013-10-09

**Authors:** Melissa J Ward, Samantha J Lycett, Dorita Avila, Jonathan P Bollback, Andrew J Leigh Brown

**Affiliations:** 1 Institute for Evolutionary Biology, University of Edinburgh, Ashworth Building, West Mains Road, Edinburgh EH9 3JT, Scotland, UK; 2 IST Austria, Am Campus 1, Klosterneuburg 3400, Austria

**Keywords:** Influenza, Evolution, Reassortment, Selection, Subtype

## Abstract

**Background:**

Reassortment between the RNA segments encoding haemagglutinin (HA) and neuraminidase (NA), the major antigenic influenza proteins, produces viruses with novel HA and NA subtype combinations and has preceded the emergence of pandemic strains. It has been suggested that productive viral infection requires a balance in the level of functional activity of HA and NA, arising from their closely interacting roles in the viral life cycle, and that this functional balance could be mediated by genetic changes in the HA and NA. Here, we investigate how the selective pressure varies for H7 avian influenza HA on different NA subtype backgrounds.

**Results:**

By extending Bayesian stochastic mutational mapping methods to calculate the ratio of the rate of non-synonymous change to the rate of synonymous change (*d*_*N*_/*d*_*S*_), we found the average *d*_*N*_/*d*_*S*_ across the avian influenza H7 HA1 region to be significantly greater on an N2 NA subtype background than on an N1, N3 or N7 background. Observed differences in evolutionary rates of H7 HA on different NA subtype backgrounds could not be attributed to underlying differences between avian host species or virus pathogenicity. Examination of *d*_*N*_/*d*_*S*_ values for each subtype on a site-by-site basis indicated that the elevated *d*_*N*_/*d*_*S*_ on the N2 NA background was a result of increased selection, rather than a relaxation of selective constraint.

**Conclusions:**

Our results are consistent with the hypothesis that reassortment exposes influenza HA to significant changes in selective pressure through genetic interactions with NA. Such epistatic effects might be explicitly accounted for in future models of influenza evolution.

## Background

The influenza A virus has its natural reservoir in wild waterfowl, who transmit it sporadically to other avian species along migratory flyways [1]. The main antigenic influenza proteins - the surface proteins haemagglutinin (HA) and neuraminidase (NA) - are each encoded by a separate RNA segment and are classified into subtypes which do not cross-react serologically. Reassortment – the exchange of genetic segments between co-infecting parental viruses during replication – leads to novel combinations of HA and NA subtypes. There are currently 16 known HA subtypes (H1-H16) and 9 known subtypes of NA (N1-N9) circulating in birds [2]. Whilst all of subtypes H1-H16 and N1-N9 can be found amongst wild waterfowl [3], viruses with certain HA/NA combinations occur frequently in nature whereas others are rarely observed [4-6]. This, combined with the failure of laboratory studies to produce viable reassortant viruses of particular subtype combinations, has led to the suggestion that there is a requirement for a functional match between the influenza HA and NA [7].

The HA and NA proteins play complementary roles in the life cycle of the influenza virus. Both HA and NA bind to host cell receptors containing sialic acid residues: HA to initiate viral entry into the host cell, and NA to permit the release of viral progeny from infected cells. Experimental studies have suggested that a fine balance between HA and NA activity must be achieved for productive viral infection [8]. Such a balance may, in fact, be more important for viral fitness than high levels of activity per se. For example, [9] showed that when artificially generated reassortant viruses of the N1 NA subtype were cultured, several (e.g. H3N1) only gave low yields. However, when the low-yield H3N1 culture was passaged, a number of changes occurred in the HA which reduced its receptor binding affinity, apparently to match that of the NA in the reassortant rather than to return to the high levels of HA activity found in the H3N8 parent virus.

Both the HA and NA proteins are thought to determine sensitivity of naturally-occurring influenza viruses to neuraminidase-inhibiting drugs (NAIs) [10]. In vitro studies have investigated genetic interactions between HA and NA in terms of NAI resistance. Evidence suggests that mutations in the HA which decrease receptor binding activity may compensate for a decrease in NA activity resulting from treatment with NAIs, thus restoring the balance between HA and NA function [7,11-13]. In addition, HA and NA mutations which individually confer low-level resistance to NAIs have been found to combine synergistically to confer resistance at a higher level [14]. Interdependence between the length of the NA stalk section and the number of HA glycosylation sites has been identified in laboratory strains [8,15] and may also have direct consequences for the transmission of influenza viruses to other host species. For example, influenza A viruses which have become established in terrestrial poultry may possess additional HA glycosylation sites, accompanied by deletions in the stalk section of their NA [16,17].

Reassortment has been implicated in the emergence of pandemic influenza viruses, including those of avian origin which were responsible for significant human mortality in the twentieth century [18,19] and the 2009 H1N1 pandemic strain [20]. Naturally-occurring reassortment events could affect the functional balance between the HA and NA proteins [7] and this could in turn affect their evolution. Whilst previous studies have investigated evolutionary rates of influenza (e.g. [21,22]), few have focused on how rates of evolution are affected by genetic interactions between segments [23].

Evolution of protein coding sequences can be quantified in terms of rates of synonymous (*d*_*S*_) and non-synonymous substitution (*d*_*N*_) and their ratio, *d*_*N*_/*d*_*S*_, following the counting-based methods of [24] and [25]. Departures from selective neutrality can be detected by a *d*_N_/*d*_S_ ratio which differs from 1. Positive selection is inferred when *d*_N_/*d*_S_ > 1. When *d*_N_/*d*_S_ < 1, it is inferred that purifying selection is acting. However, gene-wide estimates of *d*_N_/*d*_S_ which show overall purifying selection may mask a small number of sites experiencing positive selection. For example, while the overall rate of non-synonymous substitution across the influenza HA has been found to be lower than the synonymous substitution rate in birds and humans (e.g. [22,26]), evidence has been provided for positive selection at certain amino acid sites, particularly those of antigenic significance (e.g. [27-30]).

Avian influenza viruses of the H7 HA subtype present an epidemiological and economic threat on a global scale. Along with H5, H7 is the only subtype associated with the highly pathogenic form of avian influenza and has been known to cause outbreaks in domestic poultry (e.g. [17,31-33]), human infection [34-36] and even human mortality [34]. The danger posed by H7 viruses is exemplified by recent human infections with H7N9 avian influenza, which had claimed at least 37 lives in China as of 28 May, 2013, and has been associated with an estimated 36% fatality rate amongst cases admitted to hospital [37]. In particular, reassortment events between H7, N9 and H9N2 viruses have been suggested to have been important in the emergence of the outbreak-causing H7N9 lineage [38].

In this study, we adopted a Bayesian stochastic mutational mapping approach [39,40] to investigate how the association with different NA subtypes influences the evolution of the HA-encoding segment of avian influenza. Specifically, *d*_*N*_/*d*_*S*_ ratios of avian influenza H7 HA1 were evaluated for clades associated with different NA subtype backgrounds. We extended the mutational mapping approach of Nielsen [39,40] by rescaling the inferred numbers of synonymous and non-synonymous changes to calculate *d*_*N*_/*d*_*S*_. Ancestral trait mapping was used to construct a clade-model that inferred background NA subtypes for branches across the tree, and *d*_*N*_/*d*_*S*_ was averaged across all parts of the tree corresponding to a particular subtype. The ancestral trait mapping accounts for a lack of monophyly across the tree with respect to NA subtype background, which arises through repeated exposure of H7 HA to different NA backgrounds via reassortment. We find substantial differences between gene-wide *d*_*N*_/*d*_*S*_ for avian influenza H7 HA on different NA subtype backgrounds, consistent with the hypothesis that the selective pressure experienced by HA can be affected by its genetic context.

## Results and discussion

### Distribution of avian influenza H7 HA sequences

We downloaded all available unique avian influenza HA coding sequences from the NCBI Influenza Virus Resource and labelled them according to the NA subtype of the virus (see Methods). The dataset we analysed contained over 40 sequences from viruses of each of NA background subtypes N1, N2, N3 and N7. The distribution of these sequences with respect to other virus and host properties, specifically the taxonomic order of the avian host and the viral pathogenicity, was also considered (Table 1). Examination of the sequence names revealed that 71% of the sequences were known to have been isolated from terrestrial poultry and approximately 16% were from aquatic fowl. Most of the sequences from birds of the order Anseriformes were likely to have been isolated from farmed birds (isolates labelled "duck") (e.g. [41]) although a small number were known to be from wild aquatic birds. On all NA subtype backgrounds, the majority of sequences were from Galliformes, although isolates from Anseriformes were present for all subtypes (6 sequences from Anseriformes for H7N1 and H7N2; 13 for H7N3 and H7N7). Literature searching for laboratory-confirmed pathogenic status of avian influenza viruses revealed that approximately two-thirds of the sequences were from highly pathogenic (HP) viruses, although numbers of HP and low pathogenic (LP) isolates were not distributed evenly across the subtypes. For example, H7N2 viruses have only been reported in the low pathogenic form despite several years of circulation in live bird markets [42], whilst approximately half of the H7N1 isolates in the dataset were from HP viruses.

**Table 1:** Composition of avian H7 HA sequence dataset (background NA subtypes N1, N2, N3 and N7)

	**Subtype**			
**All subtypes (253)**	**H7N1 (62)**	**H7N2 (75)**	**H7N3 (69)**	**H7N7 (47)**
**Host order**				
**Ans. (38)**	Ans. (6)	Ans. (6)	Ans. (13)	Ans. (13)
**Gal. (173)**	Gal. (39)	Gal. (60)	Gal. (52)	Gal. (22)
**Pathogenicity**				
**HP (56)**	HP (20)	HP (0)	HP (20)	HP (16)
**LP (195)**	LP (42)	LP (75)	LP (49)	LP (29)
**Time-span (years)**	1934-2001	1978-2006	1963-2006	1927-2003
**Location**				
**Europe (118)**	Europe (53)	Europe (5)	Europe (25)	Europe (35)
**Asia (14)**	Asia (4)	Asia (4)	Asia (3)	Asia (3)
**Africa (4)**	Africa (3)	Africa (0)	Africa (0)	Africa (1)
**Australia (10)**	Australia (0)	Australia (0)	Australia (4)	Australia (6)
**N. America (99)**	N. America (2)	N. America (66)	N. America (29)	N. America (2)
**S. America (8)**	S. America (0)	S. America (0)	S. America (8)	S. America (0)

Numbers of H7 sequences associated with different NA subtypes, avian hosts, viral pathogenicities and years and locations of sampling are given in brackets. Note that it was not possible to determine such information for all sequences. For different avian host taxonomic orders, we use the abbreviations Ans. = Anseriformes, and Gal. = Galliformes.

For each background NA subtype, the H7 HA sequences covered a time-span of at least 25 years. There were roughly equal numbers of sequences from Eurasia and America (132 and 107 respectively), and sequences from Europe, Asia and North America were present for all four subtypes considered. The geographic spread of H7 avian influenza viruses of different background NA subtypes appeared to differ between continents. For example, 85% of the H7N1 sequences and 74% of the H7N7 sequences were from Europe, whilst 88% of the H7N2 isolates were from North America. H7N3 appeared to be the most ubiquitously sampled subtype, in terms of location, host order and pathogenicity. Overall, geographic and temporal diversity appeared to be captured in all subtypes.

### Phylogenetic analysis of avian influenza H7 HA

**Figure 1:**
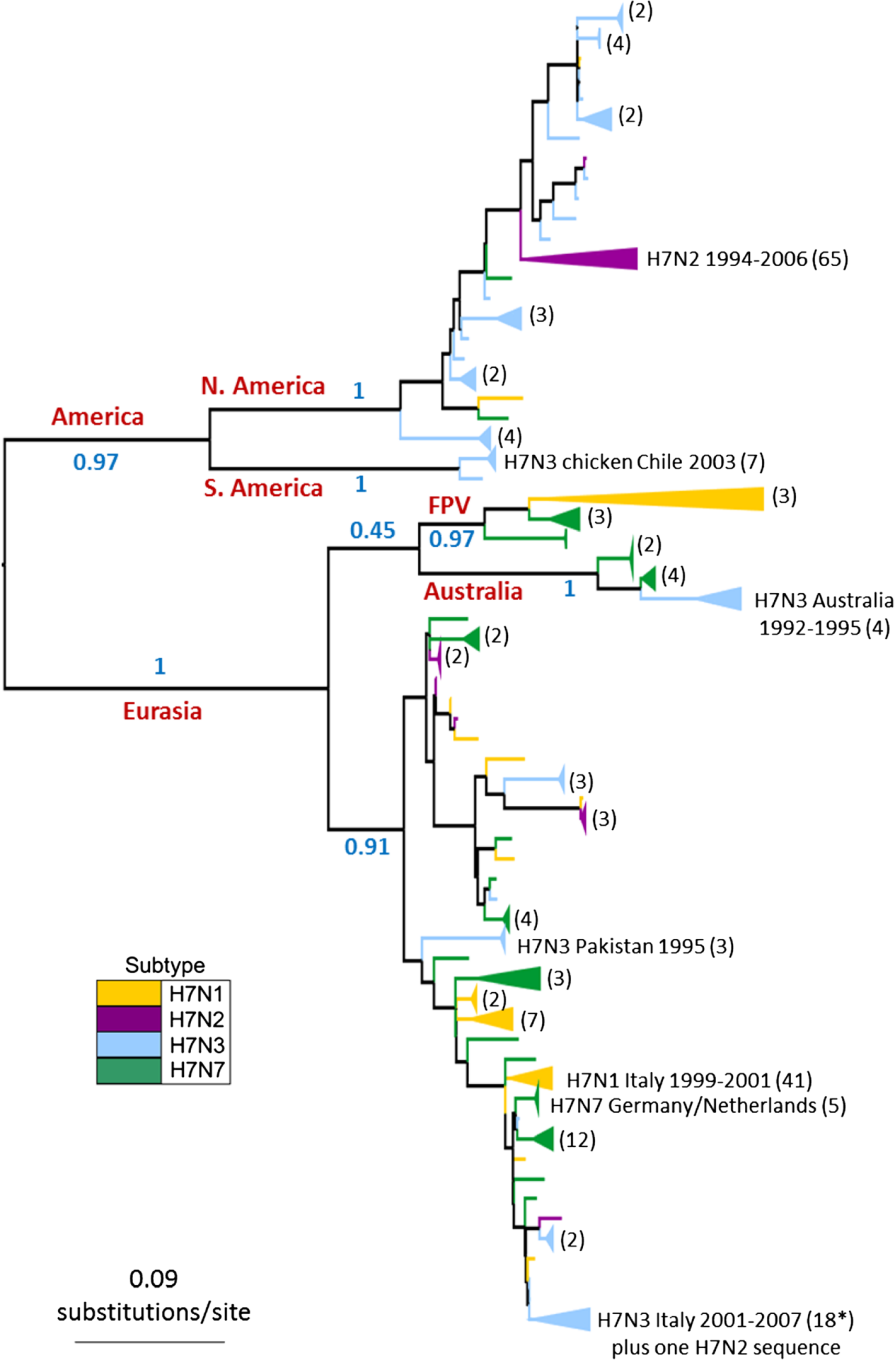
**H7 HA1 phylogeny.** The tree was inferred using the PhyML software under the GTR + Γ model of DNA substitution, with 6 rate categories. 1000 bootstrap replicates were performed. Major geographical lineages are labelled in red and bootstrap support values (proportion of bootstrap replicates) for major clades are labelled in blue. An H15 sequence was used as an outgroup, but was removed in this figure for the purpose of presentation. Lineages are coloured by the background NA subtype of the virus at the tips of the tree, and clades of sequences of the same subtype have been collapsed for the purpose of presentation (numbers of sequences in collapsed clades are given in brackets). Note: FPV = 'fowl plague virus’, a term used to describe H7 avian influenza viruses isolated in the 1920s-1940s.

Phylogenetic trees constructed for the avian influenza H7 HA1 coding region revealed a split into major geographical lineages which was consistent between maximum likelihood (ML) and Bayesian phylogenetic methods (Figure 1 and Additional file 1: Figure S1 respectively). The major lineages corresponded to viruses sampled in (a) Europe, Asia, Africa and Australasia (the 'Eurasian’ lineage: bootstrap support in ML tree = 100) and (b) North and South America (the 'American’ lineage: bootstrap support = 97%). The existence of Eurasian and American lineages has previously been identified in avian influenza H7 HA [43-45], as well as in other HA subtypes and different gene segments [1,46]. We observed a split in the American clade into North American and South American sequences (bootstrap support of 100% for both clades), which has also previously been suggested [47].

Within the Eurasian clade, the Australian isolates formed a clade with 100% bootstrap support. The maintenance of a distinct Australasian lineage of H7 avian influenza within the Eurasian clade, with continued reassortment of different NA subtypes onto the H7 HA, has recently been reported [44]. The phylogenetic position of early European fowl plague viruses (FPV) as a sister lineage to the Australian clade has been observed in other studies [43,44,48] and was observed in our ML and MrBayes phylogenies, although both methods appeared to have difficulty in placing this clade (which could account for the relatively low posterior probability observed for the Eurasian clade in the MrBayes consensus tree). Following other evolutionary studies [22], we excluded the FPV sequences from our mutational mapping analysis of evolutionary rates, since they have been highly cultured and may show artificially high rates of molecular change.

On a smaller geographic scale, H7 HA sequences from within avian influenza outbreaks, such as the Italian H7N1 outbreak of 1999–2000, clustered together. The observation that H7 HA sequences from viruses with different NA subtype backgrounds were distributed across the tree, rather than forming distinct clades, is indicative of repeated reassortment between H7 HA and NA of different subtypes. Avian H7 HA sequences did not cluster into distinct lineages corresponding to HP or LP viruses, or viruses from avian hosts of orders Anseriformes or Galliformes.

### Comparison of selection in H7 avian influenza HA on different NA subtype backgrounds

**Figure 2:**
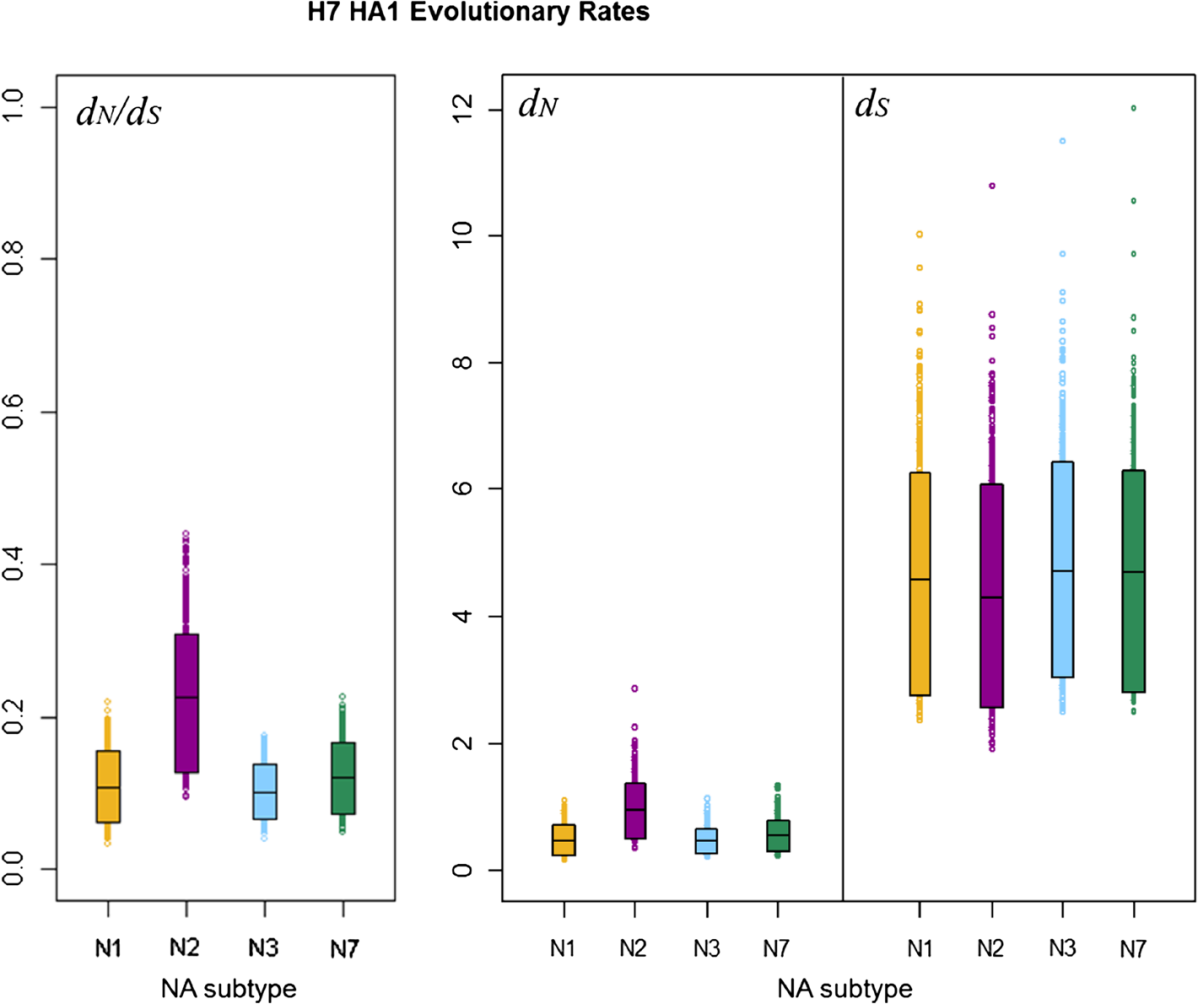
**90% HPD plots for H7 HA1 evolutionary rates, split by viral NA subtype.** The boxes show the limits of the narrowest interval containing 90% of the estimates. The horizontal lines inside the boxes indicate the location of the mean for each subtype. Individual points shown outside the boxes are values which lie below the lower limit, or above the upper limit, of the 90% HPD interval. For each subtype, values for *d*_*S*_ are the number of synonymous changes per synonymous site, scaled by the total branch lengths in the tree sample for lineages corresponding to that subtype. Similarly, *d*_*N*_ is given in terms of the number of non-synonymous changes per non-synonymous site, scaled by the total branch lengths in the tree sample for lineages corresponding to that subtype.

We used stochastic mutational mapping [39,40,49] (see Methods) to infer mutational histories for the 1000 avian influenza H7 HA1 MrBayes phylogeny samples. Estimates of *d*_*N*_ and *d*_*S*_ averaged across sites in the influenza HA1 were calculated for parts of the phylogenies corresponding to NA background subtypes N1, N2, N3 and N7 as described in Methods. This allowed the selective pressure on H7 influenza HA1 to be compared across different NA subtype backgrounds. Uncertainty in the mutational mapping process was accounted for by simulating, and averaging over, 10 mutational histories for each of the 1000 posterior phylogeny samples. The rate of synonymous substitution (*d*_*S*_) was substantially higher than the rate of non-synonymous substitution (*d*_*N*_) for avian influenza H7 HA1 on all background NA subtypes (Figure 2), with no overlap between the 90% highest posterior density (HPD) intervals for *d*_*N*_ and *d*_*S*_. Lower rates of non-synonymous substitution than synonymous substitution resulted in gene-wide *d*_*N*_/*d*_*S*_ estimates which were substantially less than one for all NA subtype backgrounds, indicating an overall pattern of purifying selection across the HA1. This is in line with previous studies [28-30], which have suggested that the influenza HA is conserved overall.

For all 1000 MrBayes phylogeny samples, the average *d*_*N*_ estimate across all HA1 sites for a given NA background was plotted against the *d*_*S*_ value for that tree sample (Additional file 1: Figure S2). This indicated that a phylogeny sample with a higher rate of synonymous substitution would also have a higher rate of non-synonymous substitution, although the rate of synonymous substitution was not an exact predictor of the corresponding non-synonymous substitution rate. It may be observed that, whilst the same *d*_*S*_ value would lead to a similar expected *d*_*N*_ for background NA subtypes N1, N3 and N7, there was little overlap between the *d*_*N*_ values on the N2 background and on backgrounds N1, N2 and N3, with the *d*_*N*_ values for N2 appearing to be higher than for the other NA background subtypes.

For each background NA subtype, the HA1-wide *d*_*N*_ value for each tree sample was divided by the *d*_*S*_ value for that tree sample, to obtain 1000 HA1-wide posterior estimates of the *d*_*N*_/*d*_*S*_ ratio on each of NA backgrounds N1, N2, N3 and N7 (Table 2). Plots of the HPD intervals for *d*_*N*_, *ds* and *d*_*N*_/*d*_*S*_ allowed posterior distributions of evolutionary rates to be visualised for H7 HA lineages associated with different NA subtypes (Figure 2). We observed similar means and 90% HPD intervals for *d*_*S*_ across all NA subtype backgrounds. However, for both *d*_*N*_ and *d*_*N*_/*d*_*S*_, the mean of the H7N2 distribution lay above the upper 90% HPD limit of the distributions for the other NA background subtypes (N1, N3 and N7). The means for *d*_*N*_ and *d*_*N*_/*d*_*S*_ for background NA subtypes N1, N3 and N7 lay below the lower limit of the 90% HPD interval for H7N2, although a small amount of overlap was observed between the lower 90% HPD limit of the distribution for H7N2 and the upper 90% HPD limit for the other subtypes.

**Table 2:** **Average *d***_***N***_**/*d***_***S ***_**across the H7 avian influenza HA1 on different NA backgrounds**

**Subtype**	**Mean *d***_***N***_**/*d***_***S***_	**Lower 90% HPD limit for *d***_***N***_**/*d***_***S***_	**Upper 90% HPD limit for *d***_***N***_**/*d***_***S***_
H7N1	0.107	0.063	0.156
H7N2	0.226	0.126	0.309
H7N3	0.102	0.067	0.137
H7N7	0.120	0.074	0.168

For each background NA subtype, the average *d*_*N*_/*d*_*S*_ across the HA1 coding region was obtained for each MCMC sample by first averaging over mutational mapping replicates on that tree, then calculating average values for *d*_*N*_ and *d*_*S*_ across all HA1 sites. Within tree samples, the site-averaged *d*_*N*_ was divided by the site-averaged *d*_*S*_ for that NA subtype, to obtain 1000 posterior estimates of the *d*_*N*_/*d*_*S*_ ratio for each NA subtype background.

In the absence of differences in synonymous substitution rates between the subtypes, the elevated rate of non-synonymous substitution across the avian influenza HA1 in H7N2 lineages led to the apparent increase in *d*_*N*_/*d*_*S*_ for H7N2 compared to H7N1, H7N3 and H7N7. In order to compare posterior distributions of evolutionary rates for H7 HA1 on different NA subtype backgrounds, randomised pairing of sampled rate estimates on different NA backgrounds was performed (see Methods). For arbitrary background NA subtypes A and B, the proportion (denoted p) of the randomly paired samples for which the rate for subtype A was greater than for subtype B (the top value in each cell), or less than for subtype B (the bottom value in each cell), was reported (Table 3). For example, p = 0.05/0.95 would mean that the value for subtype A was greater than for subtype B in 5% of pairings, and less than for subtype B in 95% of pairings. A split at least as extreme as 0.05/0.95 in either direction was interpreted as a substantial difference in the location of the distributions for the two subtypes.

**Table 3:** Comparing evolutionary rates for H7 avian influenza HA1 on different NA subtype backgrounds

**Comparison**	***d***_***N***_**/*d***_***S***_	***d***_***N***_	***d***_***S***_
H7N1-H7N2	0.021465	0.048604	0.577697
	0.978535	0.951396	0.422303
H7N1-H7N3	0.540547	0.503311	0.467995
	0.459453	0.496689	0.532005
H7N1-H7N7	0.373000	0.356954	0.468392
	0.627000	0.643046	0.531608
H7N2-H7N3	0.991065	0.965327	0.389154
	0.008935	0.034673	0.610846
H7N2-H7N7	0.962234	0.907221	0.390056
	0.037766	0.092779	0.610846
H7N3-H7N7	0.317627	0.340218	0.501494
	0.682733	0.659782	0.498506

The proportion of randomised pairings of posterior rate samples for which the value for the first subtype in the comparison, minus the value for the second subtype in the comparison, was greater than 0 (top value in each cell) and less than 0 (bottom value in each cell) is reported. Similar distributions would be indicated by the difference being greater than 0 (likewise less than 0) in approximately 50% of pairings. Differences in the location of the distributions would be indicated by a more extreme split in one direction.

For all NA subtype comparisons, the distributions of paired differences for *d*_*S*_ were roughly centred on zero (i.e. approximately 50% of the paired differences were greater than zero, and 50% less than zero), indicating no substantial differences between the distributions, as suggested by the HPD interval plot. However, the pairwise difference comparisons indicated an elevated rate of non-synonymous change in H7N2, leading to a substantially higher *d*_*N*_/*d*_*S*_ for H7N2 than for the other subtypes (split of p = 0.979/0.021 against H7N1; p = 0.991/0.009 against H7N3; p = 0.962/0.038 against H7N7).

Our results for the ordering of *d*_*N*_/*d*_*S*_ values across H7 HA1 on different NA subtype backgrounds are consistent with the point estimates obtained by a previous study [22] which was based upon the single likelihood ancestor counting (SLAC) method [50]. The results from [22] could not be statistically compared between subtypes and did not account for uncertainty in the phylogenetic or mutational history. Furthermore, estimating *d*_*N*_/*d*_*S*_ separately for H7 HA datasets corresponding to different background NA subtypes, as was carried out in [22], implicitly assumes that the tree of all H7 HA sequences should split into distinct clades according to background NA subtype. Our phylogenetic analysis, along with previous studies (e.g. [43]), has shown that H7 HA sequences are not monophyletic with respect to viral NA subtype. It is therefore possible that error might be introduced into *d*_N_/*d*_S_ estimates from datasets corresponding to individual NA subtype backgrounds, by incorrectly assuming that ancestral lineages were associated with a particular NA subtype.

### Comparison of avian influenza H7 HA1 *d*_*N*_/*d*_*S*_ by virus pathogenicity and avian host

**Figure 3:**
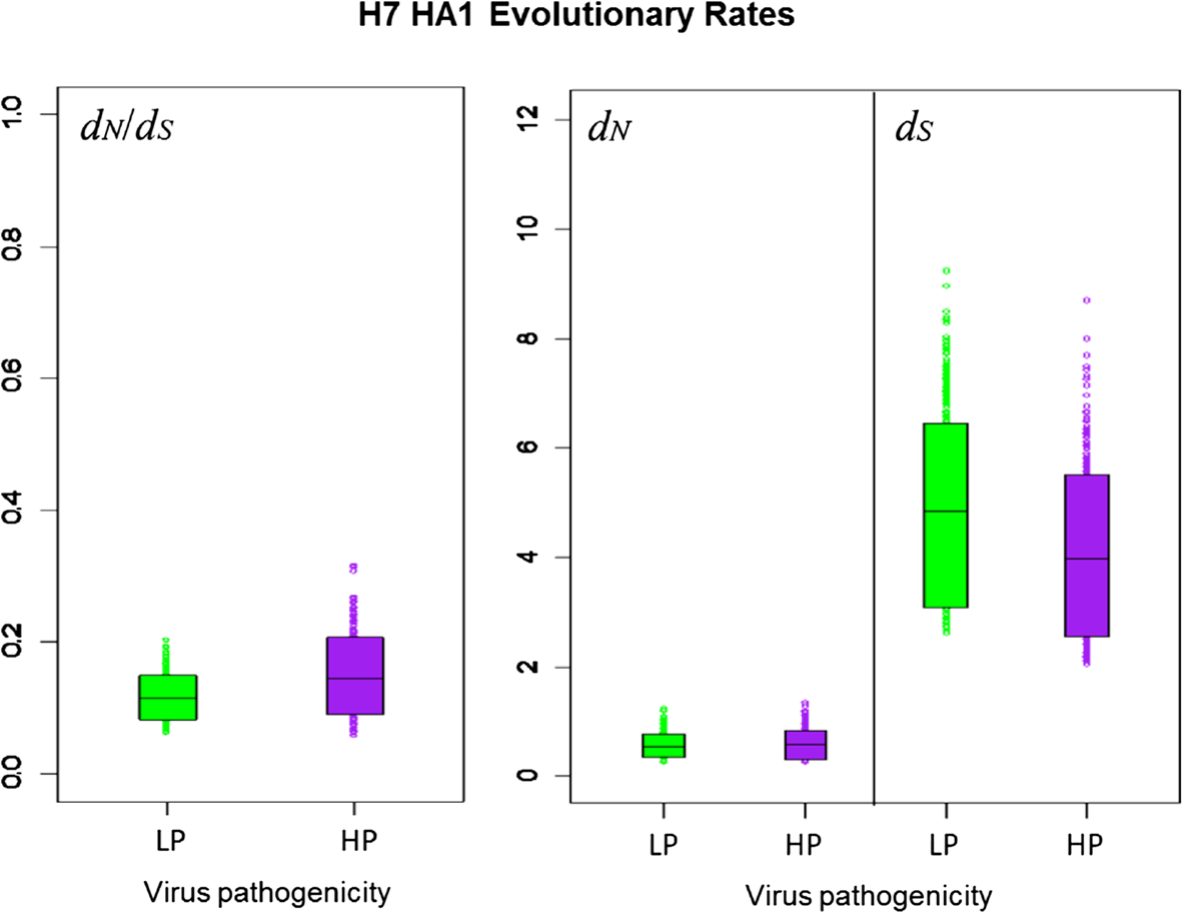
**90% HPD plots for H7 HA1 evolutionary rates, split by virus pathogenicity.** The coloured boxes show the limits of the narrowest interval containing 90% of the posterior estimates. The horizontal lines inside the boxes indicate the location of the mean for highly pathogenic (HP) or low pathogenic (LP) viruses. The similarity in evolutionary rates for HP and LP viruses can be observed from the overlap in the distributions and the location of the means of the distribution for HP viruses within the 90% HPD limits of the corresponding LP distribution and vice versa.

**Figure 4:**
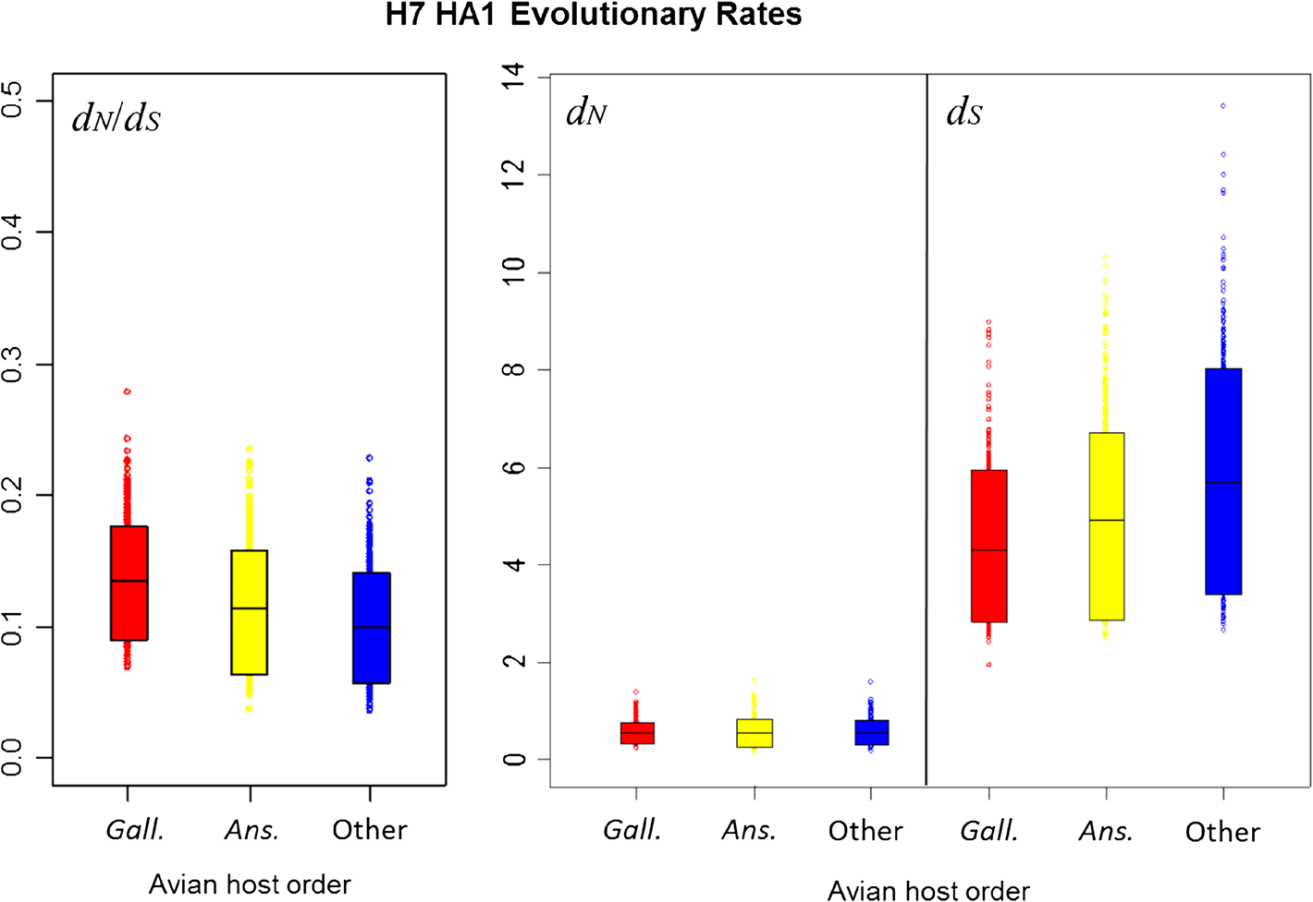
**90% HPD plots for H7 HA1 evolutionary rates, split by avian host order.** The means and HPD limits for *d*_*N*_/*d*_*S*_ and rates of synonymous and non-synonymous substitution were similar for anseriform (Ans.), galliform (Gal.) and other avian hosts. This indicated that the taxonomic order of the avian host from which influenza viruses were isolated did not have a significant effect on evolutionary rates or selective pressure experienced by the virus.

The distribution of the avian influenza H7 HA sequences we analysed was not uniform across NA subtypes in terms of virus pathogenicity or avian host (Table 1). We therefore carried out further mutational mapping analyses to assess whether differences in avian host or viral pathogenicity might have confounded the comparisons of evolutionary rates of H7 HA on different NA subtype backgrounds. Evolutionary rates *d*_*N*_, *d*_*S*_ and their ratio, *d*_*N*_/*d*_*S*_, were compared for lineages corresponding to highly pathogenic (HP) and low pathogenic (LP) avian influenza viruses, and for viruses isolated from Anseriformes (ducks, geese etc.), Galliformes (turkeys, chickens etc.) and other avian hosts (see Methods for details). As may be observed from the means and 90% HPD intervals for *d*_*N*_/*d*_*S*_ (Figure 3 and Table 4) and the randomised pairing analysis for comparing distributions (Table 5), *d*_*N*_, *d*_*S*_ and *d*_*N*_/*d*_*S*_ did not differ substantially between HP and LP lineages, indicating that viral pathogenicity did not have a discernible effect on the average selective pressure experienced across H7 avian influenza HA1. Likewise, no substantial difference was observed in the distributions of evolutionary rates between lineages corresponding to viruses sampled from avian host orders Anseriformes or Galliformes (Figure 4, Table 6 and Table 7). We also investigated the relationship between the proportion of sequences from terrestrial poultry (Galliformes) and *d*_*N*_/*d*_*S*_ for each background NA subtype and did not find a significant correlation between them (*p* = 0.9167, Additional file 1: Figure S3), although the power to detect a significant effect would be low, due to the existence of just four data points.

**Table 4:** **Average *d***_***N***_**/*d***_***S ***_**across H7 avian influenza HA1 for lineages corresponding to different viral pathogenicities**

**Virus pathogenicity**	**Mean *d***_***N***_**/*d***_***S***_	**Lower 90% HPD limit for *d***_***N***_**/*d***_***S***_	**Upper 90% HPD limit for *d***_***N***_**/*d***_***S***_
HP	0.146	0.092	0.207
LP	0.115	0.082	0.150

Stochastic mutational mapping was used to calculate *d*_*N*_/*d*_*S*_ along lineages corresponding to viruses of high pathogenicity (HP) and low pathogenicity (LP) for 1000 MCMC tree samples, in an analogous manner to that described for comparisons by background NA subtype.

**Table 5:** Comparing H7 avian influenza HA1 evolutionary rates along lineages classified by viral pathogenicity

**Comparison**	***d***_***N***_**/*d***_***S***_	***d***_***N***_	***d***_***S***_
HP-LP	0.763821	0.519682	0.26037
	0.236179	0.480318	0.73963

Evolutionary rate distributions were compared for highly pathogenic (HP) and low pathogenic (LP) lineages in an analogous manner to that described for different background NA subtypes.

**Table 6:** **Average *d***_***N***_**/*d***_***S ***_**across H7 avian influenza HA1 for lineages corresponding to different avian host orders**

**Avian host order**	**Mean *d***_***N***_**/*d***_***S***_	**Lower 90% HPD limit for *d***_***N***_**/*d***_***S***_	**Upper 90% HPD limit for *d***_***N***_**/*d***_***S***_
Anseriformes	0.113	0.065	0.158
Galliformes	0.135	0.091	0.177
Other	0.100	0.057	0.141

Stochastic mutational mapping was used to calculate *d*_*N*_/*d*_*S*_ along lineages corresponding to viruses from different avian host orders (Anseriformes, Galliformes and others) for 1000 MCMC tree samples, in an analogous manner to that described for comparisons by background NA subtype.

**Table 7:** Comparing H7 avian influenza HA1 evolutionary rates along lineages classified by avian host order

**Comparison**	***d***_***N***_**/*d***_***S***_	***d***_***N***_	***d***_***S***_
Ans. - Gall.	0.293355	0.443505	0.647044
	0.706645	0.556495	0.352956
Ans. - other	0.637318	0.482577	0.336128
	0.362682	0.517423	0.663872
Gall. - other	0.821002	0.541115	0.213498
	0.178998	0.458885	0.786502

Evolutionary rate distributions corresponding to lineages from avian hosts of orders Anseriformes (Ans.) and Galliformes (Gal.) were compared in an analogous manner to that described for different background NA subtypes.

### Site-by-site analysis of H7 HA1 *d*_*N*_/*d*_*S*_ on different NA subtype backgrounds

**Figure 5:**
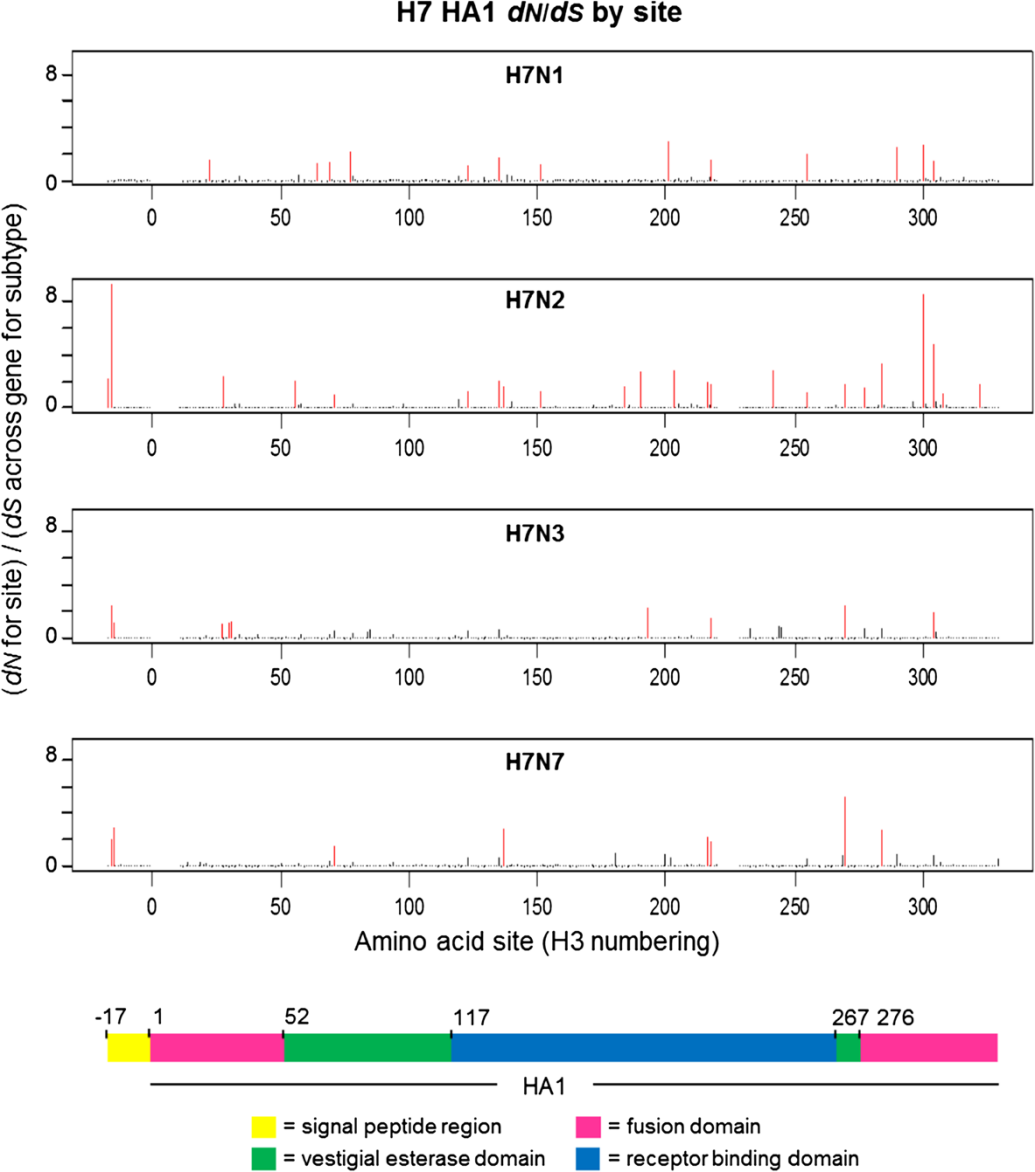
**Distribution of *d***_***N***_**/*d***_***S***_ **values across avian influenza H7 HA1 sites, on different NA subtype backgrounds.** The *d*_*N*_ value for each site was divided by the average *d*_*S*_ across all sites for that subtype to obtain a *d*_*N*_/*d*_*S*_ value for each site on each background NA subtype. Sites with *d*_*N*_/*d*_*S*_ > 1, i.e. under putative positive selection, are highlighted in red. Sites under putative positive selection were distributed across the HA1 for all background NA subtypes. Although there is some variation between NA backgrounds in terms of the sites under putative positive selection, there is also some commonality between the subtypes (see Additional file 1: Table S1). A coloured key is provided, which indicates the HA1 domain: fusion (pink), vestigial esterase (green) or receptor binding (blue). The signal peptide region is indicated in yellow.

**Figure 6:**
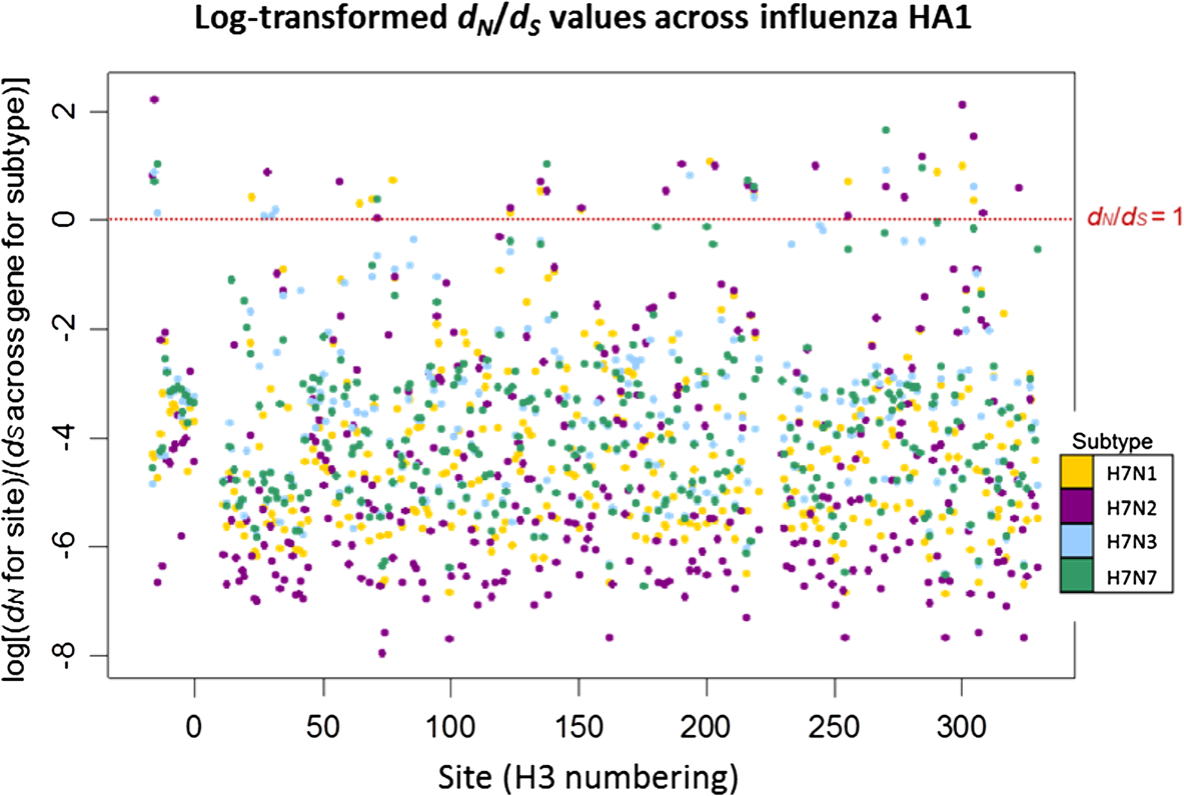
**Log(*d***_***N***_**/*d***_***S***_**) values across avian influenza H7 HA1 sites, on different NA subtype backgrounds.** The natural logarithm of the *d*_*N*_/*d*_*S*_ values from was taken, so that sites with log(*d*_*N*_/*d*_*S*_) > 0 corresponded to *d*_*N*_/*d*_*S*_ > 1, and sites with log(*d*_*N*_/*d*_*S*_) < 0 corresponded to *d*_*N*_/*d*_*S*_ < 1 (the value log(*d*_*N*_*/d*_*S*_) = 0, i.e. *d*_*N*_*/d*_*S*_ = 1, is shown as a dotted red line). The *d*_*N*_/*d*_*S*_ values for each site are colour coded according to the background NA subtype. Codon sites correspond to the H3 numbering.

Estimates of *d*_*N*_ and *d*_*S*_ at individual H7 HA1 codon sites were calculated separately for each NA background subtype in order to investigate the process driving differences in selective pressure between H7 HA1 on an N2 NA background, compared to an N1, N2 or N3 background, and to identify sites under putative positive selection. Of the 329 codon sites studied, the vast majority (more than 96% of sites on all NA subtype backgrounds) had a mean *d*_*N*_/*d*_*S*_ ratio of less than 1. A small number of sites were identified as being under putative positive selection, i.e. with mean *d*_*N*_/*d*_*S*_ > 1 across mutational mapping replicates and phylogeny samples, and such sites were distributed across the HA1 sub-segment (Figure 5, Figure 6 and Additional file 1: Table S1). The domain in which each site with mean *d*_*N*_/*d*_*S*_ > 1 was observed was recorded. Sites under putative positive selection were observed in all domains: the signal peptide region, which directs the HA protein to the virion surface; the fusion domain (also known as the membrane-proximal domain), which fuses the HA protein to the rest of the virion; the receptor binding domain, which binds to sialic acid receptors in host cells, and the vestigial esterase domain, whose metabolic role is redundant but which has been speculated to play some part in membrane fusion activity of modern-day influenza viruses [51].

The largest number of sites under putative positive selection was observed on the N2 NA background (23 sites under putative positive selection, out of the 329 sites considered). This was approximately twice the number of sites with a mean *d*_*N*_/*d*_*S*_ > 1 on N1, N3 or N7 backgrounds (13, 9 and 8 sites respectively). When the largest 50 mean *d*_*N*_/*d*_*S*_ values across the HA1 codon sites were ordered by magnitude for each NA background subtype, the *d*_*N*_/*d*_*S*_ value on the N2 background was higher than the *d*_*N*_/*d*_*S*_ value of that rank on all other NA subtype backgrounds (Additional file 1: Figure S4a). The large *d*_*N*_/*d*_*S*_ values observed at individual codon sites for H7 HA1 on the N2 NA background would have led to the elevated HA1-wide *d*_*N*_/*d*_*S*_ observed on the N2 NA background; however, H7N2 also had many of the smallest *d*_*N*_/*d*_*S*_ values out of the different subtypes at individual amino acid sites (Figure 6, Additional file 1: Figure S4b and Figure S5). For all NA subtype backgrounds, sites with mean *d*_*N*_/*d*_*S*_ > 1 were observed in each of the fusion, vestigial esterase and receptor binding domains.

Although high *d*_*N*_/*d*_*S*_ values were observed at two sites in the signal peptide region of H7 HA on NA backgrounds N2, N3 and N7, no sites with mean *d*_*N*_/*d*_*S*_ > 1 were observed for the H7 HA signal peptide region on the N1 NA background. The signal peptide region appears to have been considered in previous gene-wide or HA1-wide calculations of *d*_*N*_/*d*_*S*_ (e.g. [22,28]), and the values we have reported across the alignment encompass the signal peptide and HA1. Note that we observed the same general pattern of average *d*_*N*_/*d*_*S*_ across sites for H7 avian influenza on different NA backgrounds (i.e. a higher *d*_*N*_/*d*_*S*_ when H7 HA was on an N2 NA background than on an N1, N3 or N7 NA background) when averaging across just the HA1 coding region, i.e. excluding the signal peptide region (data not shown).

Some commonality was observed between the H7 HA1 sites with mean *d*_*N*_/*d*_*S*_ > 1 on different NA subtype backgrounds. One site (site 218 in H3 numbering) had mean *d*_*N*_/*d*_*S*_ > 1 on all four NA subtype backgrounds; 3 amino acid sites had mean *d*_*N*_/*d*_*S*_ > 1 on 3 out of the four NA subtype backgrounds and 10 sites had mean *d*_*N*_/*d*_*S*_ > 1 in two out of the four background NA subtypes (Additional file 1: Table S1). Site 218 has been linked with receptor-binding specificity [52-54] and thus high levels of non-synonymous change at this site could signify a move towards viruses which are capable of infecting other host species.

Of the 75 H7N2 HA1 sequences studied, 66 were from viruses circulating in the North American live bird markets between 1994 and 2006, or from the many avian influenza outbreaks they seeded in commercial poultry in the Northeast United States during this period [41,55]. It may also be noted that 88% of the North American H7N2 sequences possessed a deletion of 8 amino acids at the HA receptor binding site, and a recent study has put forward the idea that non-synonymous changes might have occurred in the HA to maintain functionality [56]. This would be compatible with our observation that a large number of sites with mean *d*_*N*_/*d*_*S*_ > 1 were found in the receptor binding domain for H7 HA on the N2 NA background (Figure 5 and Additional file 1: Table S1). If the elevated level of non-synonymous change only applied to H7N2 HA1 lineages associated with the receptor binding site deletion then our results could also be compatible with this hypothesis. It is possible that molecular changes at, or adjacent to, other sites in the receptor binding region (for example, the elevated *d*_*N*_/*d*_*S*_ that we observed in H7N2 at sites 216 and 218 – H3 numbering) could be compensating for the HA deletion. Although this could indicate co-evolution at sites within the HA, again this could be to restore HA activity levels to match those of the NA.

H7N2 was the most common avian influenza subtype isolated from the North American live bird markets between 1994 and 2006 [57,58], garnering attention as a potential source for a human pandemic virus [35,59] after it proved capable of causing limited human infection [60,61]. North American H7N2 viruses isolated between 2002 and 2003 were found to exhibit increased affinity towards human-like *α*-2,6-linked sialic acid receptors [62] which has also been associated with adaptation to certain terrestrial birds, such as chickens and quails [63-65]. While (like other known H7N2 avian influenza lineages) North American H7N2 only presented in a low pathogenic form, molecular evidence suggested a step-wise accumulation of basic amino acids at the North American H7N2 HA cleavage site towards those observed in highly pathogenic viruses [41]. An elevated level of non-synonymous change amongst circulating avian influenza viruses could signify a heightened risk of molecular changes occurring which would increase the pathogenicity of the virus, or its ability to infect new species and become transmissible amongst humans. Although H7N2 avian influenza appeared to have been eradicated from domestic poultry in North America by mid-2006 [66], such findings might be particularly pertinent if the strain re-emerges.

### Advantages of stochastic mutational mapping for calculating *d*_*N*_/*d*_*S*_

Our stochastic mutational mapping method for calculating the *d*_*N*_/*d*_*S*_ ratio provides many advantages for investigating selective pressure in influenza HA on different NA subtype backgrounds in the presence of reassortment. By using the rescalings described in Methods, we are able to estimate rates of synonymous substitution (*d*_*S*_) and non-synonymous substitution (*d*_*N*_), rather than merely counting the number of synonymous or non-synonymous changes along branches [39,49]. Also, estimating *d*_*N*_ and *d*_*S*_ separately allowed us to attribute differences in the *d*_*N*_/*d*_*S*_ ratio to underlying differences in the non-synonymous or synonymous rate. Our method also enabled us to estimate *d*_*N*_ and *d*_*S*_ along parts of the HA tree corresponding to different NA subtype backgrounds, despite sequences from viruses with different NA subtypes being distributed across the tree; this does not require the introduction of additional model parameters, but merely summarizes the relevant lineages. Finally, our rescalings allowed *d*_*N*_ and *d*_*S*_ to be compared between clades of different sizes and divergence.

Bayesian methods for phylogenetic inference and mutational mapping provide an advantage over parsimony and maximum-likelihood methods since they naturally accommodate uncertainty in the phylogenetic reconstruction (by considering multiple tree and model samples) and the mutational history (by sampling multiple histories for each site in each phylogeny sample). Failing to account for phylogenetic uncertainty can lead to artificially narrow confidence intervals for estimating substitution rates [40]. We note that, whilst the topologies and relative branch lengths are consistent between our maximum likelihood and Bayesian phylogenies, the MrBayes trees had longer branch lengths. This is likely to be due to a known artefact of MrBayes [67]; however, our *d*_*N*_/*d*_*S*_ estimates for H7 HA are consistent with those from a previous study [22] which used different phylogenetic inference methods.

Another advantage over parsimony is that non-parsimonious maps are not automatically excluded. Using parsimony to minimise the number of mutations required to produce the observed pattern in the data can lead to an underestimate in substitution rates, perhaps by a factor of over 20%, and can also bias *d*_*N*_/*d*_*S*_ estimates by underestimating the number of synonymous changes in scenarios where synonymous mutations occur more frequently than non-synonymous mutations [40].

In addition to the ability to use a collection of trees and sample multiple mutational histories, our mutational mapping method also possessed advantages over the PAML maximum likelihood software [68,69]. Although PAML can be used to estimate *d*_*N*_/*d*_*S*_ along the branches of a phylogeny [70,71], its use in our study would have led to an over-parameterised model with very little power for statistical testing using likelihood ratio tests, since parameters would be estimated for each branch in the tree. Furthermore, with stochastic mutational mapping we did not have to pre-specify branches with potentially positively-selected sites, which is a requirement of the branch-site models in PAML. In addition, PAML assigns *d*_*N*_/*d*_*S*_ values for branches to a pre-determined number of rate classes (bins), which would lead to a loss of precision compared to the stochastic mutational mapping approach. Mutational mapping also records the timings of mutations across the tree, which we have used in calculating evolutionary rates, whereas existing maximum likelihood methods do not.

### Evolutionary implications

Assuming that all synonymous changes are essentially neutral, *d*_*S*_ is independent of the effective size (*N*_*e*_) of the population and is simply the mutation rate [72], although synonymous rates in RNA viruses can be affected by the virus’ secondary structure [73]. Our finding that *d*_*S*_ for H7 influenza HA1 did not vary across different NA subtype backgrounds therefore suggested that the mutation rate was constant for H7 HA1 across NA subtype backgrounds.

Under non-neutral models of evolution, differences in selective pressure could lead to differences between substitution rates [72]. Since non-synonymous changes in the HA1 coding region are likely to be non-neutral, the elevated *d*_*N*_ observed for avian influenza H7 HA1 on an N2 NA subtype background might be explained by a number of scenarios. Firstly, selection could be acting to fine-tune the functional HA-NA balance of H7 HA on an N2 NA background following reassortment. Secondly, a burst of positive selection could have occurred in the H7N2 lineages, which is not a consequence of the N2 NA background, but instead a consequence of an unrelated, co-varying factor such as avian host, demographic scenario, or an interaction with another gene segment. Thirdly, a relaxation of selective constraint could have taken place when H7 HA was exposed to the N2 NA background. The results of this study do not definitively distinguish between such scenarios and causality cannot be inferred. However, whilst *d*_*N*_/*d*_*S*_ > 1 was observed in a larger number of HA1 sites on the N2 NA background than on N1, N3 or N7 backgrounds, at many sites the N2 viruses also had the lowest *d*_*N*_/*d*_*S*_ values out of all NA subtype backgrounds (Figure 6 and Additional file 1: Figure S4b) and this is not indicative of an overall relaxation of selective constraint. One explanation for the observed pattern of site-by-site *d*_*N*_/*d*_*S*_ values could be a larger effective population size in HA for the H7N2 viruses, which would allow selection to act more effectively in removing deleterious mutations, leading to a reduction of variation at some sites.

The results presented in this study are consistent with the hypothesis that reassortment exposes HA to significant changes in selective forces via association with different NA subtypes. However, establishing a causal relationship between background NA subtype and differences in evolutionary rates of HA is not straightforward. Mutational mapping analyses excluded underlying differences in evolutionary rates between viruses of different pathogenicity, or between different avian host orders, as causative factors in the elevated *d*_*N*_/*d*_*S*_ observed in H7N2 avian influenza HA1. Nonetheless, other differences between the environments from which sequences were isolated may have influenced the selective pressure experienced. For example, it has been suggested that long term evolution in commercial poultry, which are not the natural reservoir of avian influenza, could lead to accelerated rates of evolution and the accumulation of point mutations in viruses in the live bird markets [74,75].

Although we cannot exclude prolonged circulation of avian influenza viruses in non-natural avian hosts as a factor in observing an elevated *d*_*N*_/*d*_*S*_ for H7 HA on an N2 NA background, it can be noted that 66% of the H7N1 sequences we analysed were sampled during an outbreak of LP and HP H7N1 avian influenza in domestic poultry in Italy, and that the elevated *d*_*N*_/*d*_*S*_ did not appear to extend to this subtype background. However, Italian H7N1 sequences were sampled over a period of less than two years, compared to over 12 years for H7N2 in the North American live bird markets. The effect of continuous circulation amongst non-natural avian hosts on selective pressure could be investigated in H5N1 avian influenza, which is endemic in the live bird markets of East Asia [76]. Given detailed information about the origin of the avian hosts from which viruses were collected, *d*_*N*_/*d*_*S*_ could also be compared along lineages corresponding to wild or domestic avian hosts.

Future studies could investigate rate variation along individual branches of the H7 HA1 phylogeny to determine whether the elevated *d*_*N*_/*d*_*S*_ extends to all lineages on the N2 NA subtype background (for example in both Eurasia and North America), or whether it is localised to particular parts of the tree (for example, to a particular geographical location such as the North American live bird markets, or specifically after transmission to a new avian species e.g. [77]). Further analyses could also consider whether the elevated *d*_*N*_/*d*_*S*_ observed for H7N2 HA1 also extends to other segments, for example whether the NA for these viruses showed higher levels of non-synonymous change than the NA sequences for the H7N1, H7N3 or H7N7 viruses. Other investigations could consider interactions with other influenza proteins, such as the matrix protein, with which the HA and NA both interact closely. The precise nature of the genetic changes which take place when HA is placed in a novel NA background (or vice versa) could also be explored in the laboratory using reverse genetics experiments, to provide an insight into how the balance between HA and NA activity is regulated.

Future influenza modelling studies could explicitly incorporate genetic interactions between segments, rather than assuming that their evolution is independent. Such effects might be included in extensions to frameworks such as that of Zhang et al. [78], who model the impact of reassortment on the dynamics of novel human influenza strains. Although much modelling work has focused on human influenza rather than avian influenza, a recent study suggested that evolutionary changes mediating the HA-NA functional balance were an important determinant of the transmissibility of the 2009 H1N1 pandemic influenza strain [79], thus our result might find application in models of the emergence and spread of zoonotic influenza strains in human populations.

## Conclusions

Reassortment of avian influenza segments creates novel combinations of influenza genes and repeatedly exposes segments to different genetic backgrounds. Our study has shown that the selective pressure experienced by the influenza HA can vary depending upon the genetic context in which a segment finds itself. In this case, the average *d*_*N*_/*d*_*S*_ across avian influenza HA1 of subtype H7 differed according to the background NA subtype of the virus. Observed differences in selective pressure could not be accounted for by differences in the pathogenicity of the virus, or the taxonomic order of the avian host from which it was sampled. We believe that future influenza modelling studies could incorporate epistatic interactions between gene segments, for example when considering the impact of reassortment on the emergence dynamics of novel strains.

## Methods

### Avian H7 HA dataset

All available complete H7 avian influenza nucleotide sequences for the HA protein-coding region were downloaded from the NCBI database ( http://www.ncbi.nlm.nih.gov) [80] and labelled according to the corresponding NA subtype of the virus. Sequences were screened for identity and, in the case of identical sequences, only one such isolate was included. Only NA subtypes for which there were more than 20 sequences were analysed – these subtypes were N1 (62 sequences), N2 (75 sequences), N3 (69 sequences) and N7 (47 sequences) (Table 1). Sequences were also labelled according to the taxonomic order of the avian host from which the virus was isolated (Additional file 1: Table S2). Where possible, classification of the sequences into highly pathogenic (HP) or low pathogenic (LP) was made by searching the literature for studies confirming the pathogenic status of the strain using laboratory testing. Where no record of the pathogenicity of an isolate could be found, sequences were classified as HP if they possessed a motif at the HA1/HA2 cleavage region which was the same as that of a previously confirmed HP strain, in accordance with [81]. Sequences with a novel cleavage site motif which had not been previously documented as either HP or LP were not labelled by pathogenicity.

Sequence alignment was performed manually, using BioEdit [82]. The alignment of H7 HA sequences was split at the HA1/HA2 cleavage site [83] and just the HA1 coding region, which encompasses approximately two thirds of the length of the whole HA and has the major antigenic role for the virus [84], and the signal peptide region (17 amino acids immediately preceding the start of the HA1), were analysed in this study. A single breakpoint analysis [85] in the HyPhy software [86,87] found no evidence of recombination in the alignment. Investigations using the method of Xia et al. (2003) [88] and plots of transitions and transversions against genetic distance in the DAMBE software [89] found no evidence of saturation at codon positions 1 and 2; whilst there was some evidence of saturation at the third codon position, this was not severe (Additional file 1: Figure S6).

### Phylogenetic analysis

A bootstrapped phylogenetic tree (with 1000 bootstrap replicates) was constructed for the avian influenza H7 HA1 coding region using maximum likelihood inference in the PhyML software [90]. A GTR + Γ model of nucleotide substitution [91] was used, which allowed for gamma-distributed rate variation across sites. MrBayes version 3.1.2 [92,93] was used to obtain posterior samples of topologies, branch lengths and substitution model parameters for the H7 HA1 alignment. A GTR + Γ model of nucleotide substitution was again selected. An outgroup sequence, A/Australian_shelduck/Western Australia/1756/1983(H15N2) [GenBank accession number: ABB90704], was used to root the trees. H15 been shown to be the closest HA subtype phylogenetically to H7 [22].

Three independent MrBayes runs were conducted, each with Markov Chain Monte Carlo (MCMC) searching over 2,000,000 generations. Trees and parameters were sampled every 1000 generations. The Tracer software [94] was used to inspect the chain traces, which indicated that a burnin period of 1,000,000 generations was sufficient to exclude samples taken before the chains had converged. Chain traces were compared across the three runs, with similar post-burnin values in all runs. A post-burnin sample of 1000 posterior trees and sets of parameter estimates was used for the analysis of selection.

### Bayesian mutational mapping method for calculating *d*_*N*_/*d*_*S*_

Stochastic mutational mapping [39,40,95] was used to infer mutational histories (maps) using posterior phylogeny samples taken from MrBayes runs. Mutational histories describe the nature and location of molecular changes along the branches of a phylogeny (Additional file 1: Figure S7). Stochastic mutational mapping is a Bayesian approach in which mutational histories are sampled from the posterior distribution of mappings, given the observed nucleotide data.

We briefly describe here how mutational histories may be inferred for a given nucleotide site, given a known tree and values for the parameters of a nucleotide substitution model. Firstly, the fractional likelihoods for the nucleotides A, C, T and G at each internal node are calculated using Felsenstein’s pruning algorithm [96]. Next, ancestral states are sampled from the joint posterior distribution of possible states. The ancestral state at the root of the tree is simulated by stochastically sampling from the normalized fractional likelihoods (posterior probabilities) for nucleotides at the root. This is followed by sampling the remaining ancestral states of the internal nodes by a pre-order traversal. Each new node that is sampled is conditioned on both the data and the nodes already sampled. Finally, mutational histories are simulated for all lineages (between parent and child nodes) by modelling the substitution process from an ancestral node using a continuous-time Markov chain, with parameter values obtained from the Bayesian phylogenetic runs (e.g. using MrBayes). For a dataset *D*, a mutational mapping *M* has an associated probability which can be evaluated as:

$$P\left(M|D\right)=\frac{P\left(M,D\right)}{P\left(D\right)}.$$

Thus, mappings are sampled in proportion to their posterior probability. For a more detailed description see [97].

For each of the 1000 post-burnin MrBayes phylogeny and substitution model samples, 10 mutational mappings were simulated from the posterior distribution for each nucleotide site in the H7 HA1 alignment. Within each phylogeny sample and mutational mapping replicate, the mutational history of each amino acid site in the alignment was reconstructed by combining the mutational maps for the first, second and third codon positions. Branch lengths from the maps for codon positions 1 and 2 were rescaled to the branch lengths of position 3. This allowed us to identify codon substitutions and count the number of synonymous and non-synonymous changes (*C*_*s*_ and *C*_*n*_ respectively) along different parts of the tree, as well as to record their timing along the branches (Additional file 1: Figure S8).

Our method extends the basic stochastic mutational mapping approach of Nielsen [39,40] by rescaling observed numbers of synonymous and non-synonymous changes to account for differences in the evolutionary potential for synonymous or non-synonymous changes at each codon position (i.e., the number of synonymous and non-synonymous sites in a specific codon). The method also weights by the 'dwell time’ – the time along the branch spent in each codon – to account for the fact that a higher number of changes would be expected over a longer period over evolutionary time than over a shorter period. The rescalings detailed below provide an expected value of *d*_*N*_/*d*_*S*_ = 1 under selective neutrality. For each amino acid site in the alignment, estimates of the number of synonymous and non-synonymous sites were calculated for a given part of the tree as follows:

$$S_{s}=\frac{1}{V_{T}}\overset{c}{\underset{i=1}{\sum }}\overset{3}{\underset{j=1}{\sum }}s_{\text{ij}}v_{\text{ij}}$$

$$S_{n}=\frac{1}{V_{T}}\overset{c}{\underset{i=1}{\sum }}\overset{3}{\underset{j=1}{\sum }}n_{\text{ij}}v_{\text{ij}}$$

where 

*c* = number of codon intervals (distinct codon states) along a part of the tree. A new interval occurs every time there is a nucleotide change, even if it is silent, since this alters the codon state

*j* = position of nucleotide site in the codon (1, 2 or 3)

*s*_*ij*_ = proportion of changes at the *j*^th^ codon position of the codon at interval *i* which are synonymous

*n*_*ij*_ = proportion of changes at the *j*^th^ codon position of the codon at interval *i* which are non-synonymous

*v*_*ij*_ = "mutational time interval" or "dwell time". This is obtained by multiplying the substitution rate *r*_*j*_ with the length along the branch spent in each codon state. The parameter *r*_*j*_ is drawn from a gamma distribution, whose parameters were sampled during the MrBayes analysis. A value of *r*_*j*_ is sampled for each codon position (*j* = 1, 2, or 3) at the root from its respective posterior distribution and the stochastic mutational map is then sampled under this rate

*V*_*T*_ = sum across all codon positions and over all codon intervals of the *v*_ij_s, i.e. $V_{T}=\overset{c}{\underset{i=1}{\sum }}\overset{3}{\underset{j=1}{\sum }}v_{\text{ij}}.$

Together with the *v*_ij_s, this gives a time-weighted average which assigns more weight to codons with longer dwell times.

Note that, for a single codon interval, if the dwell time information is not used then our calculation of the number of synonymous and non-synonymous sites is the same as that of Nei and Gojobori [25], since our *s*_*ij*_ is equivalent to their *f*_*i*_. However, unlike the Nei and Gojobori approach, by using the dwell time weighting we accommodate variation in branch lengths which may affect the counting procedure. Note also that Nei and Gojobori used the evolutionary distance formula of Jukes and Cantor (1969) [98] to estimate the expected number of synonymous changes per synonymous site (or non-synonymous changes per non-synonymous site) from the proportions of synonymous and non-synonymous differences between pairs of sequences. However, our method samples the full nucleotide state history across the phylogeny for each nucleotide in the alignment, thus *d*_*N*_ and *d*_*S*_ may be estimated directly by counting synonymous and non-synonymous changes along branches and rescaling by numbers of synonymous and non-synonymous sites, and dwell times, as described above. In addition, we account for uncertainty in the tree and model parameters by performing our analysis across 1000 MrBayes samples.

Values of *C*_*s*_, *C*_*n*_, *S*_*s*_ and *S*_*n*_ were used in calculating synonymous and non-synonymous evolutionary rates (*d*_*S*_ and *d*_*N*_ respectively) along different parts of the phylogeny, corresponding to background NA subtypes N1, N2, N3 and N7. In order to calculate *d*_*N*_ and *d*_*S*_ for H7 HA1 on different NA subtype backgrounds, parsimony mapping was used to assign ancestral NA subtypes at internal nodes along the MrBayes phylogeny samples, based on assignments at the tips of the phylogeny (i.e., the NA subtypes corresponding to the H7 HA sequences in our dataset). This allowed branches to be classified by NA subtype: N1, N2, N3 or N7 (Additional file 1: Figure S9). Branches where a subtype could not be unambiguously assigned from a single pass of the parsimony algorithm from the tips of the tree to the root were not used in the analysis. The use of parsimony avoids the possible confounding factor of incorrect lineage classification which could arise from methods which force ancestral states to be inferred for every branch, although the exclusion of ambiguous lineages potentially results in a loss of information. *S*_*s*_ and *S*_*n*_ were calculated as described above across all branches to which a particular NA subtype had been assigned, and numbers of synonymous and non-synonymous changes were counted along those parts of the tree.

The rate of synonymous (*d*_*S*_) change and the rate of non-synonymous (*d*_*N*_) change were calculated as:

$$d_{S}=\frac{1}{T}.\frac{C_{s}}{S_{s}}$$

and

$$d_{N}=\frac{1}{T}.\frac{C_{n}}{S_{n}}.$$

Here, *T* is obtained by summing the branch lengths at all nucleotide positions in the amino acid site, with branch lengths for the first and second codon positions rescaled to the third codon position lengths (i.e. 3* sum of the third position branch lengths), for all branches in the phylogeny to which a particular NA subtype has been assigned. Rescaling by the length of the portion of the tree corresponding to each background NA subtype allowed for a comparison of evolutionary rates between clades of different sizes. This differs from the previous mutational mapping approaches of Nielsen and others [39,40,95], including those implemented in the SIMMAP software [49]. By performing these calculations upon each of the 1000 MrBayes posterior phylogeny samples, we obtained approximations to the posterior distributions for *d*_*N*_ and *d*_*S*_ for each background NA subtype, at each codon site in the H7 HA1 alignment.

### Calculating gene-wide and site-by-site *d*_*N*_/*d*_*S*_ estimates

Estimates of *d*_*N*_ and *d*_*S*_, obtained at each codon site for each background NA subtype (see Additional file 1: Table S3 for a list of sequences used in the mutational mapping analysis), were averaged over the 10 mutational mapping replicates for each phylogeny sample. Average values of *d*_*N*_ across the sites in the HA1 alignment were obtained for each NA subtype by calculating the mean of the *d*_*N*_ values across all codon sites in the alignment (and similarly for *d*_*S*_). For all 1000 MrBayes phylogeny samples, we divided the HA1-wide *d*_*N*_ estimate for a given NA subtype by the corresponding HA1-wide *d*_*S*_ value for that subtype to obtain an approximation to the posterior distribution for the HA1-wide *d*_*N*_/*d*_*S*_ for that subtype.

Estimates of *d*_*N*_/*d*_*S*_ at individual codon sites in the H7 HA1 alignment were also calculated for each NA background subtype. For each site, *d*_*N*_ and *d*_*S*_ values were averaged over the 10 mutational mapping replicates for each tree, and then averaged over the 1000 MrBayes tree samples. To calculate the *d*_*N*_/*d*_*S*_ ratio on a site-by-site basis, *d*_*N*_ for each site was divided by the average *d*_*S*_ value across the genome for that subtype. The gene-wide *d*_*S*_ was used to avoid inflation of *d*_*N*_/*d*_*S*_ values as a result of unobserved synonymous change at individual sites, and ensured that we were conservative in identifying sites under putative positive selection. Sites with a mean value of *d*_*N*_/(gene-wide *d*_*S*_) greater than one were identified as being under putative positive selection. Sites in the H7 HA alignment were converted to H3 numbering prior to being reported, as is the convention for influenza, and numbering was based upon the alignment of Nobusawa et al. [99] (sites numbered -17 to -1 for the signal peptide region and 1 to 329 for HA1). The HA1 domain in which putatively positively selected sites were found was reported, using the alignment of Yang et al. [56] in which portions of the influenza HA corresponding to the fusion domain, vestigial esterase domain and receptor binding domain were identified.

### Comparing posterior distributions of evolutionary rates

Posterior distributions of *d*_*N*_/*d*_*S*_ and rates of synonymous and non-synonymous substitution for avian H7 HA on different background NA subtypes could be visualised by plotting highest posterior density (HPD) intervals. A 100*(1-*α*)% credible interval for a posterior distribution for a parameter *θ* is any interval [*a*, *b*] in the domain of the distribution such that the posterior probability of *θ* lying between *a* and *b* is 1 – α. The highest posterior density (HPD) interval is the narrowest such credible interval. After checking the distributions for unimodality, 90% HPD intervals were calculated using the Chen and Shao algorithm [100] in the *boa* R package for the analysis of Bayesian output [101] and plotted using a custom R script (available on request). The overlap of the HPD intervals can be used as an indicator of whether the means of the distributions are significantly different.

In order to assess the overlap between posterior distributions of evolutionary rates for different background NA subtypes, the following comparison was implemented using 'distributions of differences’. For rate distributions corresponding to arbitrary NA background subtypes A and B, a comparison method was implemented as follows. Multiple pairings of evolutionary rate estimates were drawn randomly from across the 1000 posterior samples, with one observation from subtype A and one from subtype B in each pair. The proportion of pairings for which the observed rate from subtype A was greater than the observed rate from B (and vice versa) was recorded. For a null hypothesis that there is no difference between the distributions, the point of interest is where zero lies in the distribution of paired differences. If the distributions for A and B were identical then the corresponding distribution of paired differences should be centred on zero, as one would expect A > B for half of the paired samples and A<B for the other half. However, if the proportion of samples for which A>B is extremely skewed (e.g. less than 0.05 or greater than 0.95) then zero lies in the tail of the distribution of paired differences, providing evidence that the location of the distributions is different (Additional file 1: Figure S10). A total of 10^6^ random pairings were sampled for each comparison of evolutionary rate distributions; this gave similar values to systematically comparing each of the 1000 observations for one subtype with each of the 1000 observations for the other subtype. Here we report the values from the randomized pairing approach.

### Assessing the effect of host type and pathogenicity

In this study, avian H7 HA sequences were labelled according to the NA subtype of the virus and rates of evolution were calculated for lineages corresponding to different NA subtypes. In order to test whether a non-uniform distribution of host species or pathogenic viruses across different NA backgrounds could be confounding the ability to infer differences in *d*_*N*_/*d*_*S*_ between subtypes, we performed two further analyses in an analogous manner to the NA subtype analysis. These analyses involved labelling sequences and performing stochastic mutational mapping to calculate and compare *d*_*N*_/*d*_*S*_ between (a) HP and LP viruses and (b) viruses from different avian host orders. Bird orders compared were Galliformes (turkeys, chickens etc.) and Anseriformes (ducks, geese, etc.) (Additional file 1: Table S2), with all other avian host orders combined (classified as "other") due to a paucity of sequences. To further investigate the potential effect of uneven sampling of NA subtype backgrounds with respect to avian hosts, we also performed a Spearman’s rank correlation test between the proportion of sequences from terrestrial poultry and our mean *d*_*N*_/*d*_*S*_ estimate for each background NA subtype.

## Availability of supporting data

A list of GenBank accession numbers is provided (Additional file 1: Table S3) for the sequence dataset analysed in this study.

## Abbreviations

HA: Haemagglutinin; HA1: Haemagglutinin subunit 1; HP: Highly pathogenic; LP: Low pathogenic; NA: Neuraminidase.

## Competing interests

The authors declare that no competing interests exist.

## Authors’ contributions

MJW performed the phylogenetic and stochastic mutational mapping analyses, was involved in the study design and drafted the manuscript. SJL provided an initial sequence alignment, performed preliminary mutational mapping analyses and provided guidance on the study. DA performed analysis of sequence data on development-versions of the mutational mapping software. JPB wrote the mutational mapping software as an extension of his SIMMAP code, developed the rescaling method in consultation with MJW and was involved in the interpretation of results. AJLB conceived the study and provided guidance on its design. All authors read and approved the final manuscript.

## Supplementary Material

Additional file 1: Table S1 H7 HA1 sites with *dN*/*dS* > 1 in stochastic mutational analysis on different NA subtype backgrounds. **Table S2**: Classification of avian hosts of H7 influenza virus by taxonomic order. **Table S3**: H7 avian influenza sequence dataset. **Figure S1** H7 HA1 MrBayes consensus phylogeny. **Figure S2** The rate of non-synonymous substitution (*dN*) plotted against the rate of synonymous substitution (*dS*) for avian influenza H7 HA1 from viruses with different background NA subtypes. **Figure S3** Relationship between proportion of sequences from terrestrial poultry (Galliformes) and mean dN/dS for each background NA subtype. **Figure S4** Site-by-site *dN*/*dS* values across the avian influenza H7 HA1, ranked by size. **Figure S5** Histograms showing frequency of different log(*dN*/gene-wide *dS*) values across the H7 HA1 alignment for H7N1, H7N2, H7N3 and H7N7 lineages. **Figure S6** Plot of transitions (s) and transversions (v) against genetic distance for H7 HA dataset. **Figure S7** Example nucleotide mutational maps. **Figure S8** Example codon map obtained using stochastic mutational mapping. **Figure S9** Example parsimony reconstruction of background NA subtypes on a phylogeny of H7 HA sequences. **Figure S10** Testing for differences between posterior distributions of evolutionary rates for different NA background subtypes. Click here for file
