# Supplementary material for: Evolutionary interactions between haemagglutinin and neuraminidase in avian influenza
Source: BMC Evol Biol. 2013 Oct 9;13:222. doi: 10.1186/1471-2148-13-222 (PMC3854068; doi:10.1186/1471-2148-13-222)
Supplement: Additional file 1: Table S1 — H7 HA1 sites with dN/dS > 1 in stochastic mutational analysis on different NA subtype backgrounds. Table S2: Classification of avian hosts of H7 influenza virus by taxonomic order. Table S3: H7 avian influenza sequence dataset. Figure S1 H7 HA1 MrBayes consensus phylogeny. Figure S2 The rate of non-synonymous substitution (dN) plotted against the rate of synonymous substitution (dS) for avian influenza H7 HA1 from viruses with different background NA subtypes. Figure S3 Relationship between proportion of sequences from terrestrial poultry (Galliformes) and mean dN/dS for each background NA subtype. Figure S4 Site-by-site dN/dS values across the avian influenza H7 HA1, ranked by size. Figure S5 Histograms showing frequency of different log(dN/gene-wide dS) values across the H7 HA1 alignment for H7N1, H7N2, H7N3 and H7N7 lineages. Figure S6 Plot of transitions (s) and transversions (v) against genetic distance for H7 HA dataset. Figure S7 Example nucleotide mutational maps. Figure S8 Example codon map obtained using stochastic mutational mapping. Figure S9 Example parsimony reconstruction of background NA subtypes on a phylogeny of H7 HA sequences. Figure S10 Testing for differences between posterior distributions of evolutionary rates for different NA background subtypes. [file 1471-2148-13-222-S1.pdf]

H7 HA1 Site (H3 numbering)	H7N1	H7N2	H7N3	H7N7
-17		X		
-16		X	X	X
-15			X	X
22	X			
27			X	
28		X		
30			X	
31			X	
56		X		
64	X			
69	X			
71		X		X
77	X			
123	X	X		
135	X	X		
137		X		X
151	X	X		
184		X		
190		X		
193			X	
201	X			
203		X		
216		X		X
218	X	X	X	X
242		X		
255	X	X		
270		X	X	X
277		X		
284		X		X
290	X			
300	X	X		
304	X	X	X	
308		X		
322		X		

Table S1: H7 HA1 sites with d_N/d_S>1 in stochastic mutational analysis on different NA subtype backgrounds.

The d_N value for each site was divided by the average d_S across all sites for that subtype to obtain a d_N/d_S value for each site on each background NA subtype. Sites with $d_N/d_S > 1$, i.e. under putative positive selection, are reported in the table (denoted "X"). As is influenza convention, sites are numbered according to the H3 numbering for HA1 (positive sites numbers) and the peptide signal region (negative site numbers). Site numbers are coloured according to the domain: fusion (pink), vestigial esterase (green) or receptor binding (blue) following Yang *et al*. (2010) *PLoS Path*.

Avian taxonomic order	Hosts in avian H7 HA dataset
Anseriformes	Duck, goose, swan, teal, widgeon
Galliformes	Turkey, grouse, chicken, quail, pheasant, guinea-fowl, chukar
Struthioniformes	Ostrich, emu
Passeriformes	Starling, fairy bluebird, common iora
Pstittaciformes	Parrot, parakeet, conure, macaw
Charadriiformes	Gull, ruddy turnstone, shorebird/wader, red knot
Rheiformes	Rhea

Table S2: Classification of avian hosts of H7 influenza virus by taxonomic order.

The right-hand column lists the names of birds of a particular taxonomic order for which an isolate was present in the avian H7 HA sequence dataset.

1_h7n1_duck_mongolia_47_01	AB268557
2_h7n1_afristar_engq_938_79	AF149295
3_h7n1_commoniora_singapore_f89_95	AF202228
4_h7n1_fairybluebird_singapore_f92_94	AF202229
5_h7n1_africanstarling_englandq_983_79	AF202232
6_h7n1_ostrich_zimbabwe_222_96	AF202234
7_h7n1_chicken_england_71_82	AF202236
8_h7n1_fpv_egypt_45	AF202237
9_h7n1_conure_england_1234_94	AF202241
10_h7n1_parrot_england_1174_94	AF202243
11_h7n1_ostrich_southafrica_1069_91	AF202244
12_h7n1_conure_england_766_94	AF202249
13_h7n1_parakeet_netherlands_267497_94	AF202251
14_h7n1_parrot_northernireland_vf7367_73	AF202252
15_h7n1_turkey_italy_12598_99	AJ489520
16_h7n1_chicken_italy_13489_99	AJ493214
17_h7n1_chicken_italy_267_00	AJ493215
18_h7n1_turkey_italy_3889_99	AJ493466
19_h7n1_turkey_italy_4169_99	AJ493468
20_h7n1_chicken_italy_4575_99	AJ493469
21_h7n1_turkey_italy_4602_99	AJ493470
22_h7n1_turkey_italy_4603_99	AJ493471
23_h7n1_turkey_italy_3775_99	AJ493472
24_h7n1_chicken_italy_445_99	AJ580353
25_h7n1_chicken_italy_1067_99	AJ584647
26_h7n1_mallard_alberta_34_2001	CY005983
27_h7n1_turkey_italy_4169_1999	CY006037
28_h7n1_duck_nanchang_1904_1992	CY014612
29_h7n1_chicken_italy_1285_2000	CY015014
30_h7n1_turkey_italy_3675_1999	CY021413
31_h7n1_turkey_italy_1351_2001	CY021421
32_h7n1_turkey_italy_2984_2000	CY021533
33_h7n1_turkey_italy_4426_2000	CY021541
34_h7n1_chicken_italy_322_2001	CY021549
35_h7n1_duck_italy_551_2000	CY021557
36_h7n1_guineafowl_italy_155_2000	CY022661
37_h7n1_quail_italy_396_2000	CY022669
38_h7n1_chicken_italy_1082_1999	CY022677
39_h7n1_turkey_italy_977_1999	CY024754
40_h7n1_chicken_italy_2335_2000	CY024762
41_h7n1_turkey_italy_1084_2000	CY024770
42_h7n1_turkey_italy_4708_1999	CY024858
43_h7n1_turkey_italy_4295_1999	CY024874
44_h7n1_turkey_italy_3488_1999	CY024890
45_h7n1_pekinduck_italy_1848_2000	CY024898
46_h7n1_turkey_italy_4644_1999	CY025109
47_h7n1_quail_italy_4992_1999	CY025117
51_h7n1_turkey_italy_3489_1999	CY025157
52_h7n1_turkey_italy_3560_1999	CY025165
53_h7n1_turkey_italy_2715_1999	CY025173
54_h7n1_turkey_italy_2732_1999	CY025181
55_h7n1_turkey_italy_1265_1999	CY025189
56_h7n1_duck_hongkong_301_72	DQ003216
57_h7n1_ostrich_italy_2332_00	DQ991312
58_h7n1_ostrich_italy_984_00	DQ991343
59_h7n1_rhea_northcarolina_39482_1993	EF470586
60_h7n1_mallard_italy_250_02	EU158105
61_h7n1_fpv_rostock_1934	M24457
62_h7n1_ts1_1_a_fpv_rostock_1934	M24458
1_h7n2_duck_hongkong_301_1978	AB302789
2_h7n2_chicken_newyork_131425_94	AF072384
3_h7n2_turkey_newyork_44505_94	AF072386
4_h7n2_chicken_newyork_138337_95	AF072388
5_h7n2_chicken_newyork_80302_96	AF072393
6_h7n2_chicken_pennsylvania_117671_97	AF072395
7_h7n2_chicken_newyork_67773_97	AF072396
8_h7n2_turkey_pennsylvania_7975_97	AF072397
9_h7n2_chicken_pennsylvania_135521_98	AF072398
10_h7n2_quail_newyork_1398951_98	AF072399
11_h7n2_turkey_israel_ramon_79	AF202235
12_h7n2_psittacine_italy_1_91	AF202242
13_h7n2_gull_italy_6922_93	AF202248
14_h7n2_avian_ny_1183531_2001	AY240877
15_h7n2_avian_ny_7041112_00	AY240878
16_h7n2_avian_ny_742112_00	AY240879
17_h7n2_avian_ny_762473_00	AY240880
18_h7n2_avian_ny_817465_00	AY240881
19_h7n2_avian_ny_77296_00	AY240882
20_h7n2_chicken_fl_903484_01	AY240883
21_h7n2_chicken_nj_1188785_01	AY240884
22_h7n2_chicken_nj_1503837_02	AY240885
23_h7n2_chicken_nj_15124418_02	AY240886
24_h7n2_chicken_nj_158149_99	AY240887
25_h7n2_chicken_nj_608_02	AY240888
26_h7n2_chicken_ny_1190557_01	AY240890
27_h7n2_chicken_ny_1192567_01	AY240891
28_h7n2_chicken_ny_13878_98	AY240893
29_h7n2_chicken_ny_215868_99	AY240895
30_h7n2_chicken_ny_224094_99	AY240896
31_h7n2_chicken_ny_307493_00	AY240897
32_h7n2_chicken_pa_1490921_02	AY240900
33_h7n2_chicken_va_32_02	AY240906
34_h7n2_chicken_ny_1485812_99	AY240907
35_h7n2_guineafowl_ma_14808111_02	AY240908

48_h7n1_turkey_italy_4294_1999	CY025133	36_h7n2_guineafowl_nj_132469_98	AY240909
49_h7n1_turkey_italy_4617_1999	CY025141	37_h7n2_turkey_nc_11165_02	AY240910
50_h7n1_turkey_italy_4301_1999	CY025149	38_h7n2_turkey_va_55_02	AY240912
39_h7n2_turkey_va_66_02	AY240913	14_h7n3_turkey_england_63	U20462
40_h7n2_turkey_va_67_02	AY240914	15_h7n3_turkey_italy_3620_2003	CY021357
41_h7n2_chicken_nj_17206_99	AY240915	16_h7n3_turkey_italy_1010_2003	CY021365
42_h7n2_chicken_newjersey_20621_99	AY240916	17_h7n3_mallard_delaware_418_2005	CY021637
43_h7n2_chicken_ny_3572_98	AY240917	18_h7n3_turkey_utah_2472110_1995	EF470585
44_h7n2_chicken_nj_15827_99	AY240918	19_h7n3_chicken_britishcolumbia_cn7_2004	EF470587
45_h7n2_chicken_ny_13986_99	AY240919	20_h7n3_mallardduck_alb_279_1977	CY005976
46_h7n2_chicken_ny_341733_99	AY240920	21_h7n3_shorebird_delaware_22_06	EU030968
47_h7n2_chicken_ny_147142_1999	AY240921	22_h7n3_laughinggull_delaware_42_06	EU030984
48_h7n2_goose_newjersey_86003_98	AY240922	23_h7n3_turkey_italy_9742_2002	CY028660
49_h7n2_quail_ny_11430_99	AY240923	24_h7n3_turkey_italy_3829_2004	CY028676
50_h7n2_quail_pa_20304_98	AY240924	25_h7n3_gsc_chicken_b_britishcolumbia_04	AY611524
51_h7n2_avian_ny_730636_00	AY240925	26_h7n3_chicken_chile_1842404322_2002	CY015027
52_h7n2_chicken_hebei_1_2002	AY724257	27_h7n3_chicken_england_4054_2006	EF467826
53_h7n2_chicken_de_hobo_2004	AY831668	28_h7n3_turkey_italy_4479_2004	CY020581
54_h7n2_chicken_de_viva_2004	AY831669	29_h7n3_turkey_italy_4608_2003	CY021485
55_h7n2_chicken_md_minhma_2004	AY831670	30_h7n3_turkey_italy_2043_2003	CY022613
56_h7n2_dk_hongkong_293_1978	CY006029	31_h7n3_chicken_chile_176822_02	AY303630
57_h7n2_chicken_pennsylvania_143586_2002	CY014896	32_h7n3_cinnamonteal_bolivia_4537_2001	DQ525411
58_h7n2_quail_italy_4610_2003	CY021509	33_h7n3_turkey_italy_8000_2002	CY024738
59_h7n2_chukar_newyork_116531_2005	CY022765	34_h7n3_guineafowl_italy_266184_02	EU158110
60_h7n2_chicken_newyork_16330_2005	CY022821	35_h7n3_mallard_italy_199_01	EU158109
61_h7n2_chicken_newyork_212112_2005	CY022829	36_h7n3_turkey_england_192328_79	AF202245
62_h7n2_duck_newyork_212116_2005	CY022845	37_h7n3_turkey_oregon_1971	DQ870894
63_h7n2_chicken_newyork_212111_2005	CY024834	38_h7n3_turkey_oregon_1971	AB269693
64_h7n2_chukar_newyork_212117_2005	CY024842	39_h7n3_widgeon_alb_284_1977	CY005975
65_h7n2_duck_newyork_1436465_2005	CY028523	40_h7n3_blackduck_ohio_415_2001	CY020885
66_h7n2_chicken_newyork_31815_2006	CY028540	41_h7n3_turkey_italy_4130_2004	CY029913
67_h7n2_guineafowl_newyork_83911_2006	CY028572	42_h7n3_chicken_pakistan_34669_1995	CY015033
68_h7n2_chicken_newyork_83912_2006	CY028580	43_h7n3_turkey_italy_5425_2007	EU391536
69_h7n2_guineafowl_newyork_195014_2006	CY028596	44_h7n3_turkey_england_63	AF202238
70_h7n2_chicken_newyork_290474_2006	CY028628	45_h7n3_chicken_chile_4977_02	AY303634
71_h7n2_chicken_newyork_163264_2005	CY029833	46_h7n3_turkey_chile_4418_02	AY303635
72_h7n2_chicken_ny_31815_06	DQ873807	47_h7n3_turkey_italy_2987_2003	CY021493
73_h7n2_guineafowl_ny_464918_2006	DQ907528	48_h7n3_turkey_italy_3337_2004	CY021501
74_h7n2_chicken_wales_1306_2007	EF675618	49_h7n3_chicken_queensland_1994	CY022685
75_h7n2_duck_hongkong_293_78	U20461	50_h7n3_chicken_newyork_1227311_1999	AY240892
1_h7n3_chicken_newyork_147149_1999	AY240894	51_h7n3_mallard_italy_33_01	AY586411
2_h7n3_turkey_england_1963	CY015065	52_h7n3_chicken_britishcolumbia_04	AY611524
3_h7n3_turkey_italy_8912_2002	CY020605	53_h7n3_greenwingedteal_alb_228_1985	CY005978
4_h7n3_chicken_pakistan_447_95	AF202226	54_h7n3_mallardduck_alberta_435_1985	CY014587
5_h7n3_chicken_victoria_1_92	AF202227	55_h7n3_turkey_italy_2685_2003	CY028668
6_h7n3_chicken_pakistan_cr2_95	AF202230	56_h7n3_turkey_italy_3477_2004	CY028684
7_h7n3_chicken_queensland_667_95	AF202231	57_h7n3_turkey_oregon_1971	DQ870888
8_h7n3_chicken_chile_4957_02	AY303632	58_h7n3_turkey_tennessee_1_79	AB269692

9_h7n3_chicken_chile_4968_02	AY303633	59_h7n3_turkey_oregon_1971	M31689
10_h7n3_mallard_netherlands_12_2000	CY014718	60_h7n3_ruddyturnstone_nj_65_1985	CY005928
11_h7n3_turkey_minnesota_1200_1980	CY014778	61_h7n3_chicken_england_4266_2006	EF467825
12_h7n3_mallard_ohio_322_1998	CY016188	62_h7n3_turkey_italy_251_2003	CY020589
13_h7n3_bluewingedteal_ohio_658_2004	CY018901	63_h7n3_chicken_chile_4322_02	AY303631
64_h7n3_chicken_britishcolumbia_gsc_human_b_04	AY646078	22_h7n7_mallard_sweden_103_02	AY999987
65_h7n3_mallard_alberta_24_01	DQ017504	23_h7n7_mallard_sweden_104_02	AY999988
66_h7n3_mallard_alberta_24_01	DQ017513	24_h7n7_mallard_sweden_105_02	AY999989
67_h7n3_pheasant_minnesota_917_1980	CY014721	25_h7n7_mallard_sweden_106_02	AY999990
68_h7n3_chicken_victoria_224_1992	CY025077	26_h7n7_mallard_sweden_107_02	AY999991
69_h7n3_chicken_italy_270638_02	EU158111	27_h7n7_ruddyturnstone_de_2378_1988	CY005980
1_h7n7_duck_taiwan_33_1993	AB297923	28_h7n7_redknot_nj_325_1989	CY005981
2_h7n7_duck_taiwan_ya103_1993	AB297925	29_h7n7_fowl_dobson_1927	CY014992
3_h7n7_turkey_ireland_pv74_1995	AF028021	30_h7n7_starling_victoria_1985	CY024778
4_h7n7_nonpsittacine_englandq_1985_89	AF202240	31_h7n7_chicken_victoria_1976	CY024786
5_h7n7_turkey_northernireland_vf1545c5_98	AF202246	32_h7n7_chicken_victoria_1_1985	CY025069
6_h7n7_turkey_england_647_77	AF202247	33_h7n7_chicken_netherlands_03010132_03	EF015551
7_h7n7_macaw_england_626_80	AF202250	34_h7n7_mallard_italy_299_05	EU158104
8_h7n7_ostrich_southafrica_m320_96	AF202253	35_h7n7_duck_jiangxi_1742_03	EU158108
9_h7n7_chicken_germany_r28_03	AJ620350	36_h7n7_fpv_weybridge	L37794
10_h7n7_netherlands_127_03	AY338455	37_h7n7_goose_leipzig_137_8_1979	L43913
11_h7n7_chicken_netherlands_1_03	AY338458	38_h7n7_goose_leipzig_187_7_1979	L43914
12_h7n7_netherlands_219_03	AY338459	39_h7n7_goose_leipzig_192_7_1979	L43915
13_h7n7_mallard_sweden_56_02	AY999977	40_h7n7_chicken_victoria_1_1985	M17735
14_h7n7_mallard_sweden_82_02	AY999978	41_h7n7_starling_victoria_1_1985	M17736
15_h7n7_mallard_sweden_85_02	AY999979	42_h7n7_chicken_leipzig_79	U20459
16_h7n7_mallard_sweden_87_02	AY999980	43_h7n7_duck_heinersdorf_s495_6_86	U20465
17_h7n7_mallard_sweden_92_02	AY999982	44_h7n7_chicken_jena_1816_87	U20469
18_h7n7_mallard_sweden_93_02	AY999983	45_h7n7_fpv_dutch_27	Z12617
19_h7n7_mallard_sweden_94_02	AY999984	46_h7n7_chicken_ireland_1733_89	AF202239
20_h7n7_mallard_sweden_100_02	AY999985	47_h7n7_chicken_victoria_75	Z47199
21_h7n7_mallard_sweden_102_02	AY999986		

Table S3: H7 avian influenza sequence dataset.

GenBank accession numbers are provided (Note: only the HA1 sub-segment of the influenza HA was used for the phylogenetic and mutational mapping analyses. Identical sequences and early European sequences sampled between 1927 and 1945 were excluded from the mutational mapping analyses). Sequence names were re-formatted for compatibility with sequence analysis software. Sequences were downloaded from the NCBI influenza virus resource: (http://www.ncbi.nlm.nih.gov/genomes/FLU/Database/)

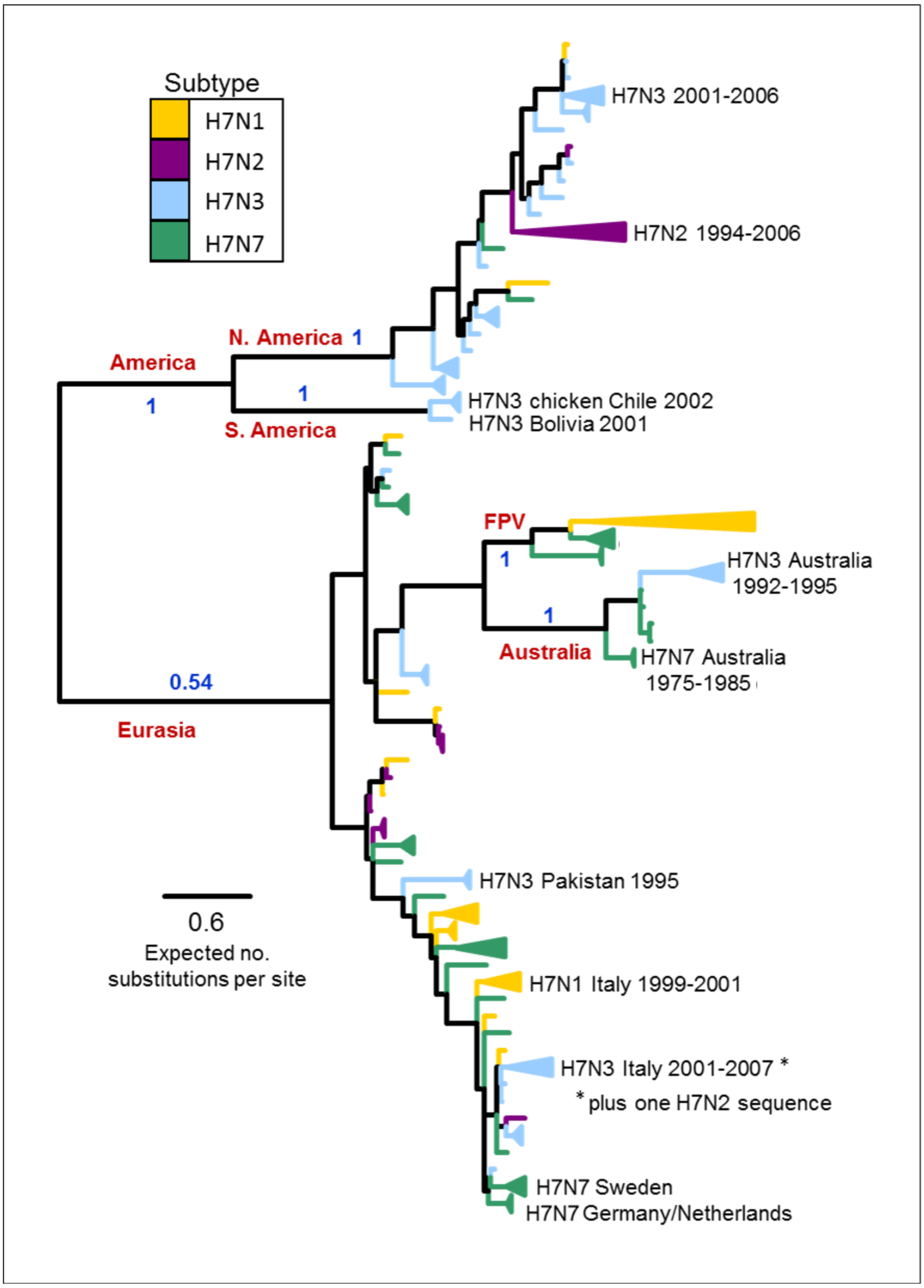

Figure S1

H7 HA1 MrBayes consensus phylogeny. The tree was inferred under the GTR + Γ model of DNA substitution, with 6 rate categories, and constructed from 1000 post-burnin MCMC phylogeny samples from MrBayes. Major geographical lineages are labelled in red and posterior probabilities of clades are labelled in blue. An H15 sequence was used as an outgroup, but was removed in this figure for the purpose of presentation. Lineages are coloured by the background NA subtype of the virus at the tips of the tree, and clades of sequences of the same subtype have been collapsed for the purpose of presentation. Note: FPV = 'fowl plague virus', a term used to describe H7 avian influenza viruses isolated in the 1920s-1940s.

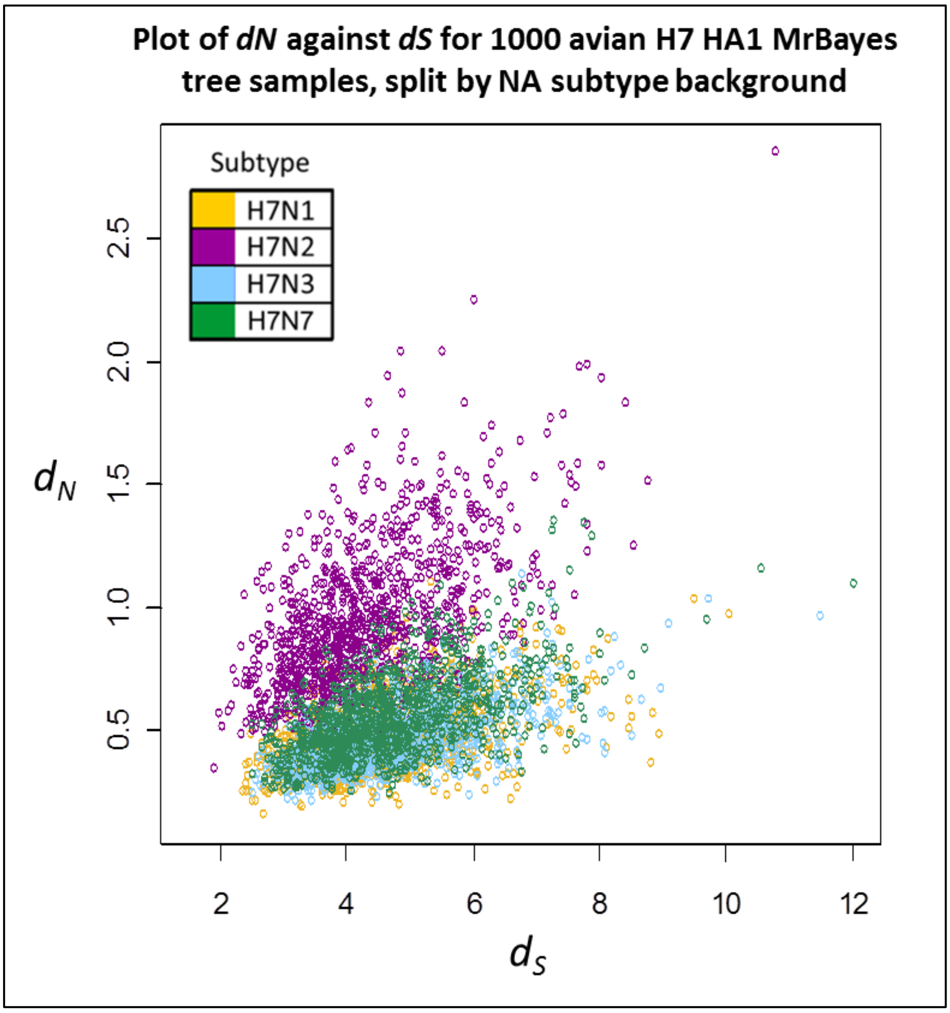

Figure S2

The rate of non-synonymous substitution (d_N) plotted against the rate of synonymous substitution (d_S) for avian influenza H7 HA1 from viruses with different background NA subtypes. For each of the 1000 MCMC tree samples from MrBayes, the value of d_N was plotted against the value of d_S for H7N1, H7N2, H7N3 and H7N7, so that the rates for different subtypes could be directly compared. It may be observed that, whilst the d_S values were similar for all four subtypes, there was little overlap between the H7N2 d_N values and those for the other subtypes. For each subtype, the linear regression line for the d_N value for a tree sample against the d_S value for the tree sample is shown.

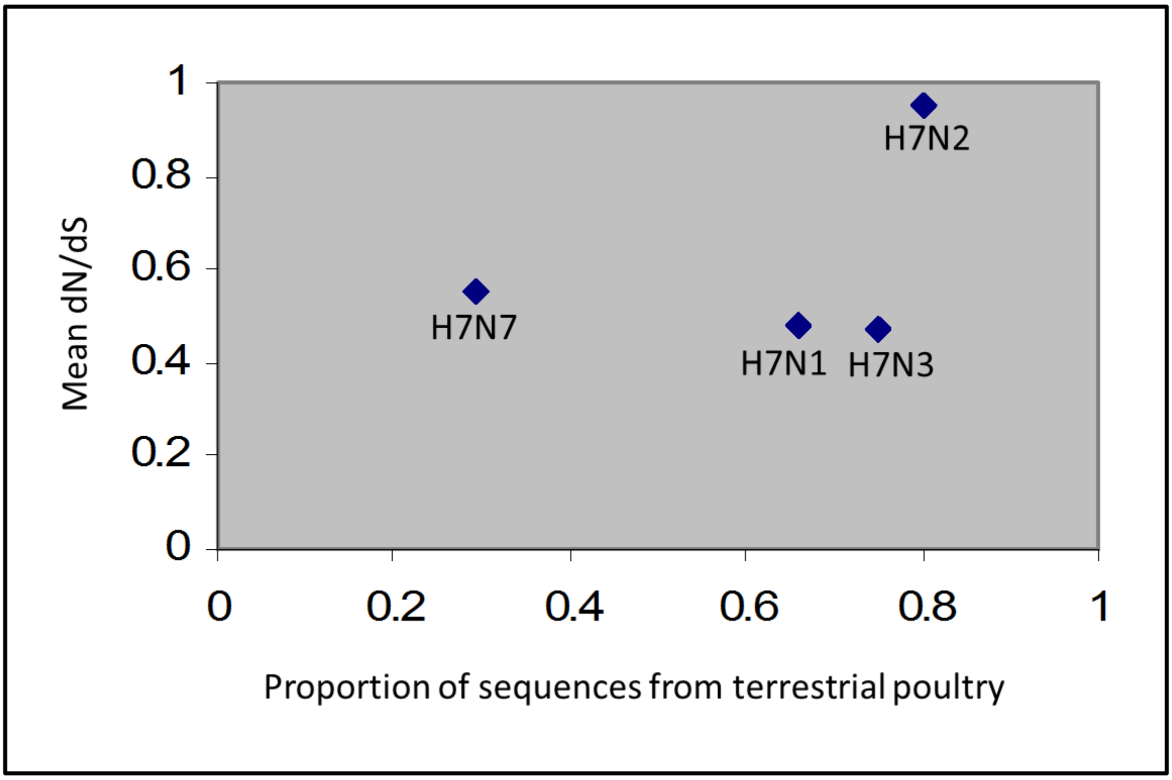

Figure S3

Relationship between proportion of sequences from terrestrial poultry (Galliformes) and mean d_N/d_S for each background NA subtype. For each background NA subtype, the proportion of sequences in the dataset which were from terrestrial poultry was plotted against the mean dN/dS for H7 avian influenza HA1, inferred by stochastic mutational mapping. A Spearman's rank correlation test did not indicate a significant correlation between d_N/d_S and proportion of sequences from terrestrial poultry (p = 0.9167).

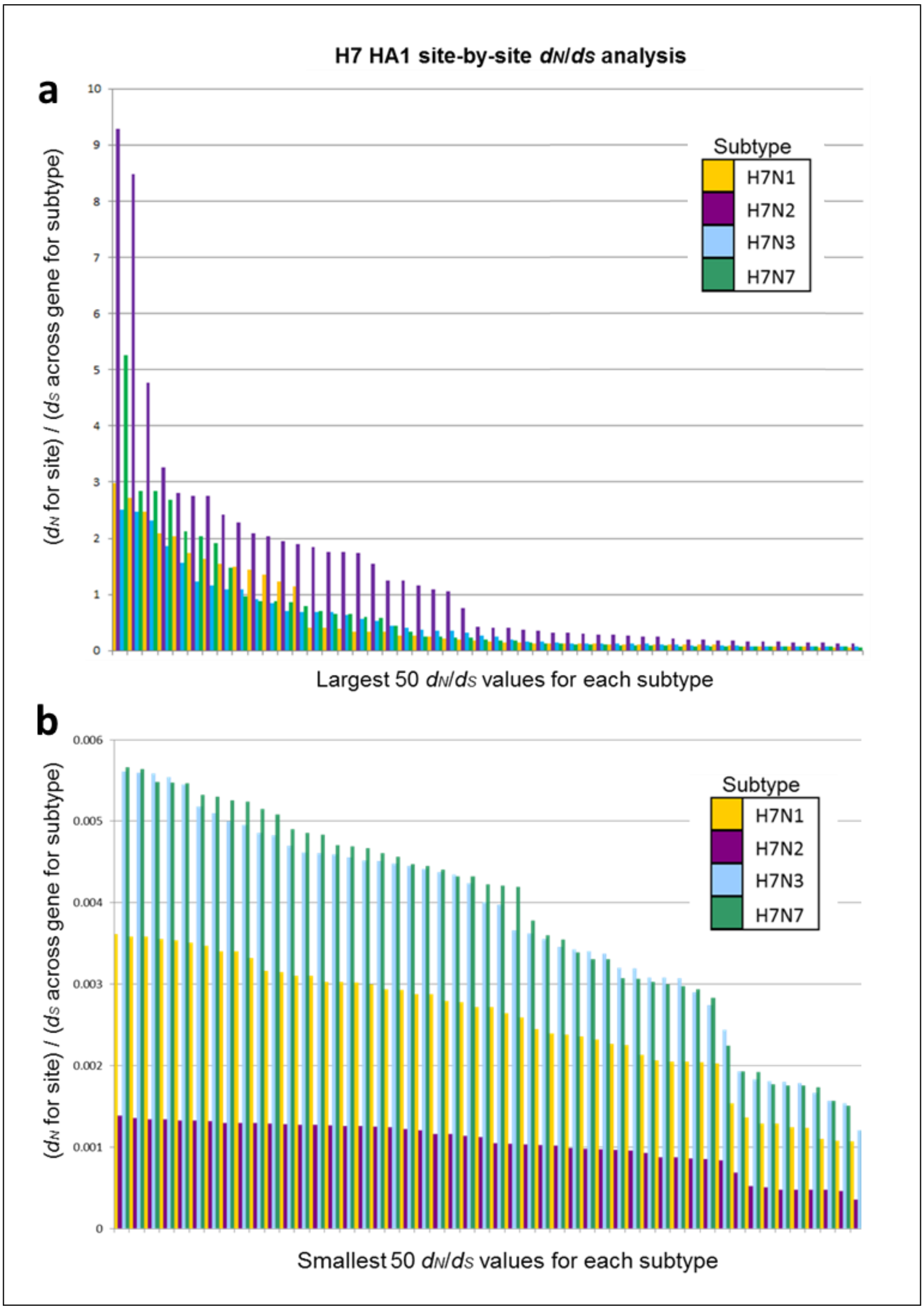

Figure S4

Site-by-site d_N/d_S values across the avian influenza H7 HA1, ranked by size. For each NA background subtype, the d_N value for each site was divided by the average d_S across all sites for that subtype. The site-by-site d_N/d_S values were ranked by size: (a) the largest 50 values were plotted for each subtype and (b) the smallest 50 values were plotted for each subtype. For all of the largest 50 d_N/d_S values, d_N/d_S on the N2 NA background was larger than the values of the same rank on the N1, N3 or N7 NA backgrounds. For all of the smallest 50 d_N/d_S values for the H7 HA1 sites, the value of d_N/d_S on the N2 NA background was smaller than the values of the same rank on the N1, N3 or N7 NA backgrounds.

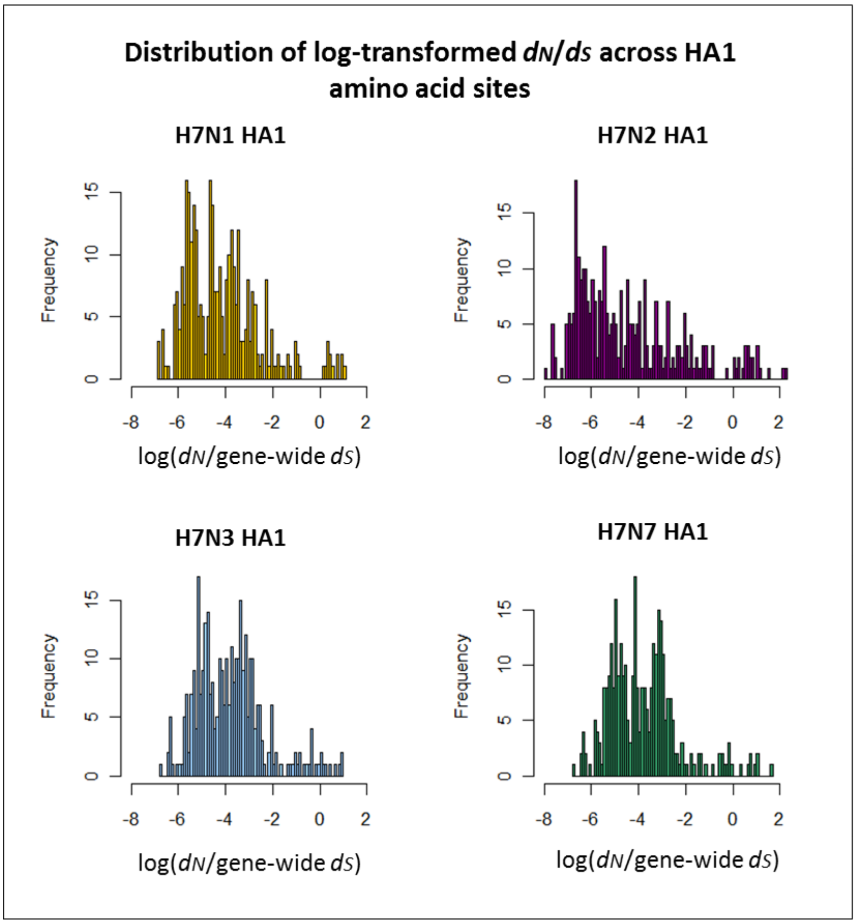

Figure S5

Histograms showing frequency of different log(d_N/gene-wide d_S) values across the H7 HA1 alignment for H7N1, H7N2, H7N3 and H7N7 lineages. Sites with log(d_N/d_S) > 0 correspond to $d_N/d_S > 1$, and sites with log(d_N/d_S) < 0 correspond to $d_N/d_S < 1$. H7N2 was the only subtype for which log(d_N/d_S) values less than -7, or greater than 2, was observed.

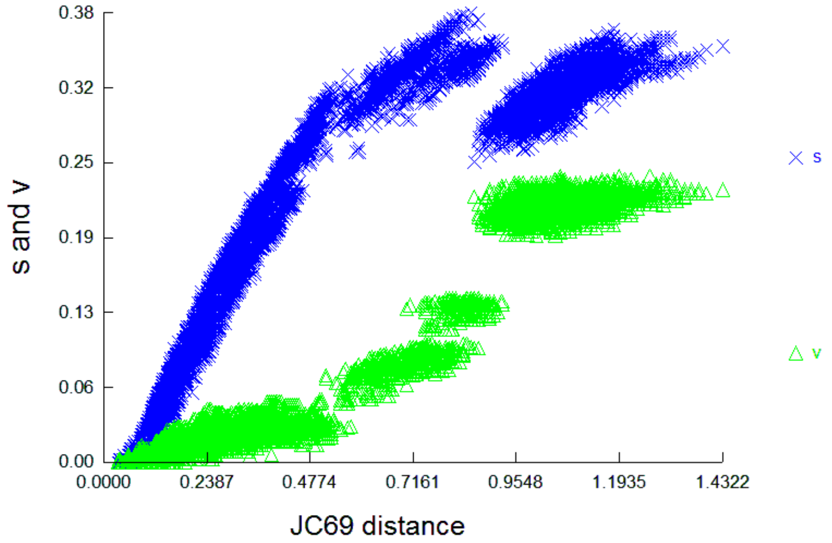

Figure S6

Plot of transitions (s) and transversions (v) against genetic distance for H7 HA dataset. Although some saturation was observed at higher genetic distances, the number of transitions remains higher than the number of transversions at all genetic distances.

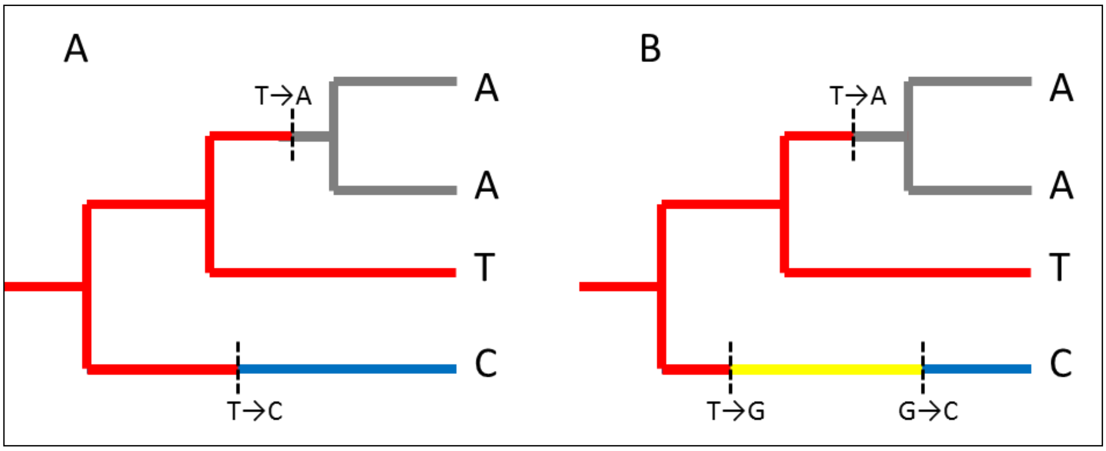

Figure S7

Example nucleotide mutational maps. Stochastic mutational mapping is used to infer mutational histories for nucleotide sites. Mutational histories report the nature and location of molecular changes along a phylogeny. Multiple mutational mappings may be sampled for each site. For example, maps (A) and (B) are both valid reconstructions for the observed pattern of variation.

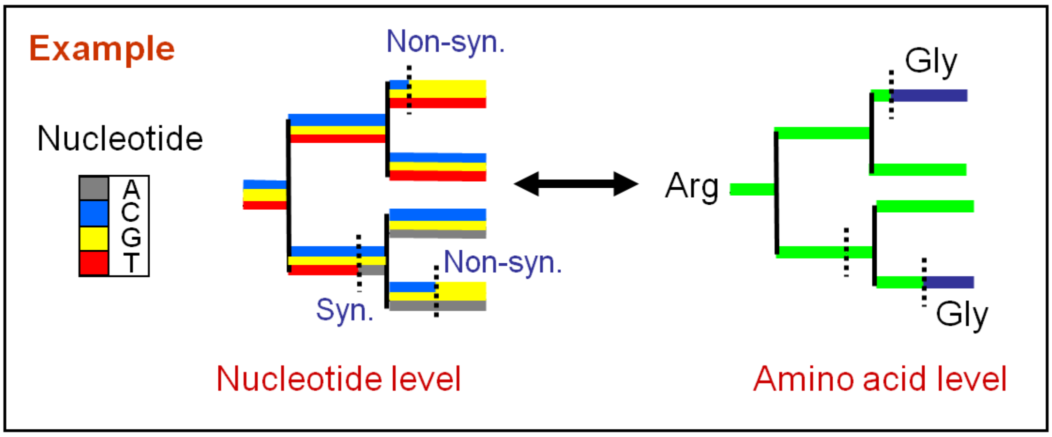

Figure S8

Example codon map obtained using stochastic mutational mapping. For each codon site, the first and second codon position nucleotide maps for a site were rescaled to the branch lengths of the third position map and combined to produce a map at the amino-acid level. Nucleotide changes could then be labelled as synonymous or non-synonymous for calculating d_N, d_S and d_N/d_S. In this example there are three nucleotide changes, one of which is synonymous (CGT → CGA) and two of which are non-synonymous (CGT → GGT and CGA → GGA).

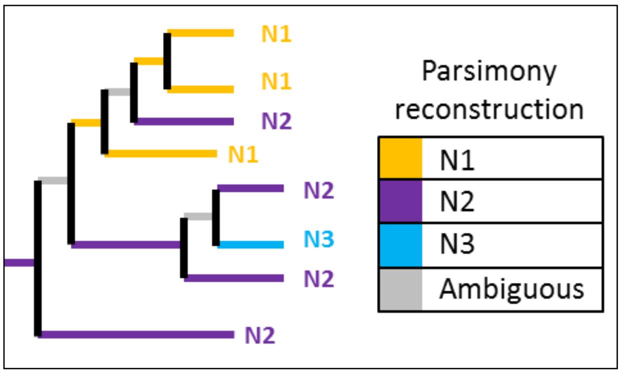

Figure S9

Example parsimony reconstruction of background NA subtypes on a phylogeny of H7 HA sequences. Branches are coloured according to the inferred ancestral subtype of the node immediately preceding them towards the tips of the tree. A single-pass algorithm was implemented, which labels some branches as ‘ambiguous’. This avoids the problem associated with erroneous assignment of subtypes in the subsequent calculation of evolutionary rates along branches associated with a particular NA subtype.

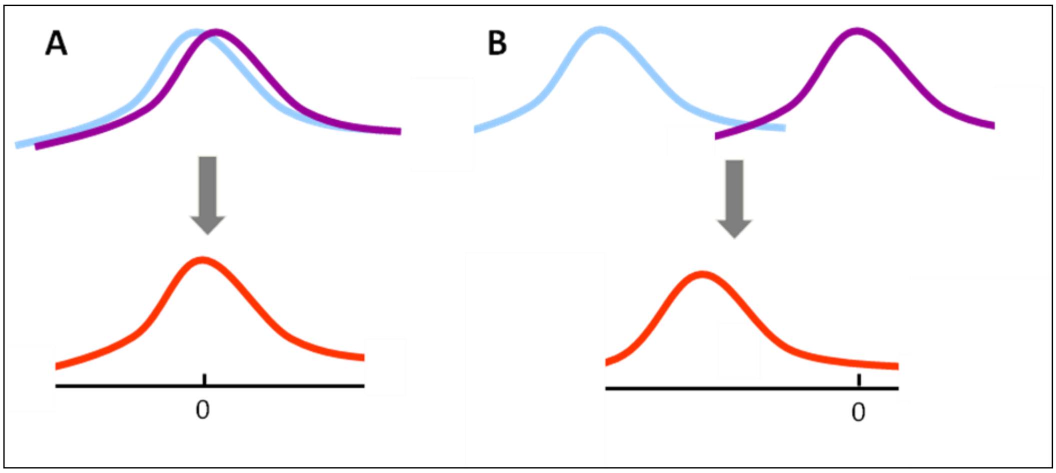

Figure S10

Testing for differences between posterior distributions of evolutionary rates for different NA background subtypes. (A) When the location of the distributions (examples shown here in blue and purple) is very similar, the distribution of differences of randomised pairings between them (shown in red) will be roughly centred on zero. **(B)** When the distributions differ in their location, the distribution of differences between randomised pairings will be skewed, with zero at one of the tail ends. The proportion of pairings lying to each side of zero thus provides a measure of the difference in location of the distributions.
